# Tumor‐Specific Delivery of CD28 siRNA via Lyso‐PC C‐16 Modified Lipid Nanoparticles Overcomes Anti‐PD‐1 Resistance by Remodeling Tumor Microenvironment

**DOI:** 10.1002/advs.76003

**Published:** 2026-06-09

**Authors:** Yangyang Chai, Keyu Wang, Jiali Fang, Shaorui Jia, Yansong Shi, Wanfeng Gao, Xinpeng Liu, Jiaqiang Li, Zenghui Cui, Yazhi Qian, Xiaosu Chen, Dan Ding, Xuetao Cao

**Affiliations:** ^1^ State Key Laboratory of Medicinal Chemical Biology Institute of Immunology College of Life Sciences Nankai University Tianjin P. R. China; ^2^ Frontiers Science Center for New Organic Matter State Key Laboratory of Medicinal Chemical Biology College of Life Sciences and Academy for Advanced Interdisciplinary Studies Nankai University Tianjin P. R. China; ^3^ Department of Immunology Center for Immunotherapy Institute of Basic Medical Sciences Chinese Academy of Medical Sciences Beijing P. R. China

**Keywords:** cancer cell CD28, cancer immunotherapy, lipid nanoparticle, PD‐L1, siRNA, tumor microenvironment

## Abstract

Uncovering novel targets that synergize with immune checkpoint blockade (ICB) is an urgent clinical priority. While cancer cell‐intrinsic CD28 facilitates immune escape by functioning as a non‐classical RNA‐binding protein to stabilize *CD274* (PD‐L1) mRNA, inhibiting this pathway in cancer cells without impairing essential T cell CD28 costimulation remains a major structural challenge. Here, we report the development of a 16:0 LPC‐modified SM102‐based lipid nanoparticle (LPC‐LNP) that exploits altered tumor lipid metabolism for highly selective cancer cell transfection. By leveraging elevated lysophosphatidylcholine acyltransferase (LPCAT) uptake mechanisms inherent to malignant cells, LPC‐LNPs efficiently deliver *Cd28* small interfering RNA directly to tumor cells while strictly avoiding T cell sequestration. In vivo administration of LPC‐LNP‐Cd28 successfully knocked down 80% of cancer cell CD28, substantially reduced PD‐L1 expression, and circumvented the off‐target immunosuppression observed with commercial lipid formulations. Consequently, targeted *Cd28* silencing reshapes the immunosuppressive tumor microenvironment, augmenting twofold CD8^+^ T cell infiltration and dendritic cell activation to extend survival in murine breast and lung cancer models to 1.3–1.5 folds. Furthermore, the combined use of LPC‐LNP‐Cd28 effectively eradicates resistance to anti‐PD‐1 therapy. This study provides a highly translatable, cancer‐specific nanomedicine platform, confirming that selectively antagonizing tumor‐intrinsic CD28 holds profound promise for advanced cancer immunotherapy.

## Graphical abstract

This study develops 16:0 LPC‐modified lipid nanoparticles (LPC‐LNPs) with cancer cell specificity by exploiting altered tumor lipid metabolism. LPC‐LNPs encapsulating *Cd28* small interfering RNA (LPC‐LNP‐Cd28) knock down cancer cell CD28 without affecting T cells, inflame the tumor microenvironment, and overcome anti‐PD‐1 resistance. These findings provide a translatable, cancer‐specific nanomedicine platform, confirming that selectively antagonizing tumor‐intrinsic CD28 holds promise for cancer immunotherapy.

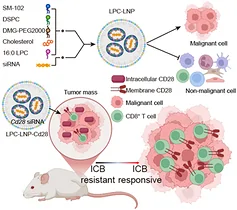

## 1 Introduction

Cancer immunotherapy, particularly the implementation of immune checkpoint blockade (ICB) targeting the Programmed Cell Death Protein 1 (PD‐1) and Programmed Cell Death Ligand 1 (PD‐L1) axis, has profoundly reformed the clinical management of multiple solid and hematological malignancies [1, 2]. By interrupting the immune‐inhibitory pathways induced by cancer cells, immune checkpoint inhibitors reinvigorate exhausted cytotoxic T lymphocytes, enabling the immune system to recognize and eradicate malignant tissues [1]. However, the therapeutic efficacy of ICB is limited by both acquired and primary resistance, with the overall objective response rate below 30% [3]. This widespread resistance is orchestrated by highly complex, dynamic alterations within the tumor microenvironment (TME), where malignant cells actively recruit immunosuppressive cells, downregulate antigen presentation machinery, and continuously evolve alternative immune evasion pathways [4]. Addressing these underlying mechanisms of immunotherapy resistance while concurrently innovating sophisticated drug delivery systems, such as advanced lipid nanoparticle (LNP) [5, 6, 7], cell membrane‐coated nanoplatforms [8], and highly specific antibody‐drug conjugates [9], is critical for the clinical translation of newly identified therapeutic targets.

CD28 is a well‐characterized and highly conserved co‐stimulatory molecule traditionally associated exclusively with the lymphoid lineage, being primarily expressed on the surface of CD4^+^ T and CD8^+^ T cells [10]. The intracellular signaling cascade initiated by CD28 engagement with its specific ligands, CD80 and CD86, on the surface of antigen‐presenting cells is an absolute requirement for robust T cell activation, sustained proliferation, and long‐term differentiation during both homeostasis and pathogenic immune responses [10, 11]. Besides, inhibition of T cell activation in TME mediated by PD‐1/PD‐L1 interaction relies on constraining CD28 signaling [12]. Anti‐PD‐1 treatment enhances T cell response in TME, a process requiring CD28 co‐stimulatory signaling [13]. Chimeric antigen receptor T (CAR‐T) cells are engineered T cells with an artificial TCR to target cancer cells, and CD28‐derived co‐stimulatory domain is frequently used in CAR‐T products to effectively activate CAR‐T cells [14]. These studies emphasize the important role of CD28 in T cell‐mediated antitumor immune response, making it a cornerstone of functional antitumoral immunity.

Despite its established, canonical role in promoting antitumoral immunity within the lymphoid compartment, recent investigation unveils a paradoxical and highly deleterious function for CD28 when it is aberrantly expressed within the malignant cellular compartment. Our group previously reported that cancer cell‐intrinsic, intracellular CD28 has been conclusively shown to directly bind and stabilize *CD274* messenger RNA [15]. In this capacity, tumor‐intrinsic CD28 abandons its traditional receptor signaling dynamics and instead functions non‐classically as a critical RNA‐binding protein. By shielding *CD274* transcripts from intracellular degradation, this non‐classical mechanism significantly promotes the continuous surface expression of PD‐L1, thereby driving cancer cell immune escape and rendering the tumor highly resistant to T cell‐mediated cytotoxicity. This unique biological mechanism positions tumor‐intrinsic CD28 as a highly potent, albeit structurally complex, target for cancer immunotherapy. However, because of the absolute physiological necessity of CD28 signaling for normal T cell survival and effector function, any systemic therapeutic strategy aimed at inhibiting CD28 must possess exquisite, flawless target‐cell specificity. Inhibiting cancer cell CD28 without inadvertently disrupting T cell CD28 necessitates the development of highly selective, targeted nanomedicine delivery platforms.

Small interfering RNA (siRNA) presents a powerful, highly specific therapeutic modality designed to trigger the precise degradation of target messenger RNA (mRNA) and execute subsequent sequence‐specific gene silencing [16, 17, 18]. However, the successful transition of siRNA therapeutics into standard clinical reality is impeded by several systemic and physiological barriers, prominently including rapid nuclease degradation in the bloodstream, poor cellular membrane permeability, and a fundamental lack of tissue‐ and cell‐specific delivery strategies [16]. Lipid nanoparticles are universally utilized to overcome these obstacles, serving as the gold standard for the encapsulation and systemic delivery of nucleic acid therapeutics [17]. Nevertheless, standardized lipid nanoparticles generally rely on passive accumulation within the tumor tissue through the enhanced permeability and retention effect, or they target hepatic tissues inherently via endogenous apolipoprotein E opsonization in the serum [5]. They typically lack the innate biochemical capacity for complex cell‐type specificity within the highly heterogeneous, densely packed TME [5]. Current experimental attempts to navigate lipid nanoparticles toward specific cellular populations have largely involved the chemical conjugation of targeting antibodies to the nanoparticle surface [19, 20, 21] or the incorporation of cell‐specific microRNA target sites within the 3‐prime untranslated regions of delivered mRNA payloads [22]. Unfortunately, microRNA modification strategies are fundamentally incompatible with siRNA delivery because siRNA lacks a 3‐prime untranslated region, and antibody conjugation exponentially increases manufacturing complexity, costs, and the risk of eliciting severe neutralizing immunogenic responses in patients.

To overcome these significant engineering obstacles, we sought to directly exploit the aberrant and highly active lipid metabolism characteristic of malignant cells to achieve passive, yet highly targeted delivery. Cancer cells ubiquitously upregulate lipid synthesis, scavenging, and remodeling pathways to sustain rapid, unregulated proliferation and constant membrane biogenesis under nutrient‐deprived conditions [23]. Within this context, the lysophosphatidylcholine acyltransferase (LPCAT) family of enzymes is significantly overexpressed across a wide variety of human malignancies, including colorectal carcinoma, hepatocellular carcinoma, and triple‐negative breast cancer [24]. Under normal physiological conditions, the Lands’ cycle utilizes LPCATs to remodel cellular membranes by reacylating lysophosphatidylcholine into mature structural phosphatidylcholine [24]. Tumors actively hijack this pathway, overexpressing LPCAT enzymes to rapidly sequester extracellular lysophosphatidylcholine species to construct new cellular membranes [25], creating a unique, exploitable metabolic sink. By systematically screening a variety of lipid derivatives for incorporation into a standard SM‐102‐based lipid nanoparticle architecture, we discovered that integrating 1‐Palmitoyl‐sn‐glycero‐3‐phosphocholine, also known as Lyso‐PAF C‐16 (16:0 LPC), drastically and permanently shifts the transfection tropism of the nanoparticle. The resulting formulation, termed LPC‐LNP, exhibited an elevated propensity to transfect highly metabolic cancer cells by exploiting their ravenous appetite for 16:0 LPC while concurrently avoiding uptake by critical peripheral and intratumoral immune populations, most notably T cells, which maintained homeostatic lipid requirements.

Herein, we presented the exhaustive structural optimization, functional in vitro validation, and robust therapeutic in vivo application of the Lyso‐PAF C‐16 modified LNP (LPC‐LNP) platform. By leveraging this cancer cell‐targeted nanocarrier to deliver *Cd28* siRNA (LPC‐LNP‐Cd28), we achieved highly specific, localized knockdown of tumor‐intrinsic CD28 in vivo. This resulted in the reciprocal, targeted downregulation of PD‐L1 without compromising the structural or functional integrity of T cell populations. Intratumoral administration of LPC‐LNP‐Cd28 successfully reinforced CD8^+^ T cell‐mediated antitumoral immunity, promoted dendritic cell (DC) activation, and significantly inhibited tumor progression across multiple aggressive murine models, including breast cancer and lung cancer. Importantly, when administered in combination with systemic anti‐PD‐1 therapy, LPC‐LNP‐Cd28 efficiently overcame preexisting immunotherapy resistance, providing a robust, highly logical, and clinically translatable methodology to deliver nucleic acid drugs to cancer cells for precision oncological genetic manipulation and cancer immunotherapy.

## 2 Results

### 2.1 Incorporating 16:0 LPC Into LNPs Improves RNA Uptake and Cancer Cell Specificity

Although the traditional four‐component LNP architecture has achieved major success in the systemic delivery of nucleic acid therapeutics, its specificity and transfection efficiency in cancer cells within the complex TME of solid tumors remain limited [6, 26, 27]. Altered cancer cell lipid metabolism, including enhanced Lands‘ cycle mediated by LPCATs, [24, 28] sheds light on designing cancer cell‐specific LNP via modifying with Lands‘ cycle‐related lipids. Firstly, we verified the specificity of Lands‘ cycle‐related geneset in public single‐cell RNA sequencing (scRNA‐seq) datasets of human breast cancers and lung cancers [29, 30, 31]. The Lands‘ cycle geneset was derived from research focusing on lipid metabolism in lung cancer, including LPCATs and other related enzymes producing indispensable substrates for Lands’ cycle [32]. The geneset scoring results indicated that cancer cells harbored higher Lands‘ cycle activity than other non‐malignant cells in TME (Figure S1A–C). However, the reverse catalytic metabolism transforming phosphatidylcholine into lysophosphatidylcholine, which was mediated by *PLA2G4A*, *PLA2G4B*, and *PLA2G4C*, was scarce (Figure S1A‐C). Furthermore, we sorted major cell subsets from established 4T1 tumors and confirmed that tumor cells expressed the highest level of *Lpcat1*, *Lpcat2*, *Lpcat3*, and *Lpcat4* in mouse TME (Figure S1D). These results suggest the applicability of utilizing the cancer cell Lands‘ cycle to develop cancer cell‐specific LNP.

**FIGURE 1:**
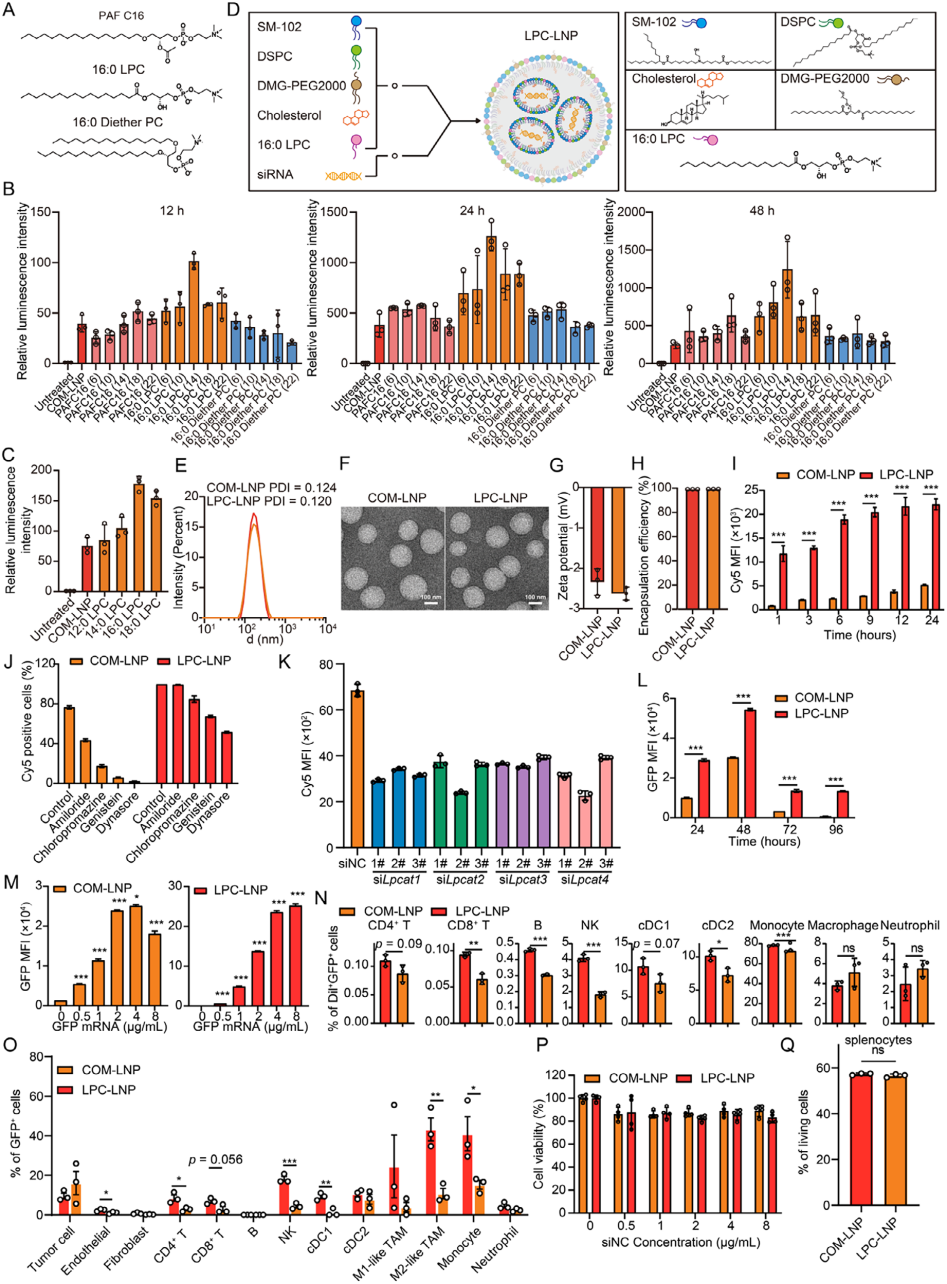
LNPs modified with 16:0 LPC improve RNA uptake and cancer cell specificity. (A) Chemical structures of the three candidate lipids used in the initial screening. (B) Initial screening of three lipids at different ratios: luciferase mRNA‐loaded LNP transfection in 4T1 cells at 12, 24, and 48 h (n = 3). (C) Screening of LPCs with different acyl chain lengths: luciferase mRNA‐loaded LNP transfection in 4T1 cells at 12 h (n = 3). (D) Schematic illustration of COM‐LNPs and LPC‐LNPs synthesis. (E‐H) Size distributions (E), TEM images (F; scale bar = 100 nm), zeta potential (G), and encapsulation efficiency (H) of LNPs (n = 3). (I) Time‐dependent cellular uptake of Cy5‐siRNA‐loaded LNPs in 4T1 cells (n = 3). (J) Endocytosis pathways of Cy5‐siRNA‐loaded LNPs in 4T1 cells (n = 3). (K) Cellular uptake of Cy5‐siRNA‐loaded LNPs in 4T1 cells with *Lpcat1*, *Lpcat2*, *Lpcat3* or *Lpcat4* knockdown using 3 individual siRNAs (n = 3). (L) Time‐dependent transfection efficiency of GFP mRNA‐loaded LNPs in 4T1 cells (n = 3). (M) Dose‐dependent transfection efficiency of GFP mRNA‐loaded COM‐LNPs (left) and LPC‐LNPs (right) in 4T1 cells (n = 3). (N) Transfection efficiency in different splenic cell subsets treated with DiI‐labeled LNPs encapsulating GFP mRNA for 24 h (n = 3). (O) Transfection efficiency in different cell populations in tumor microenvironment 24 h after intratumoral injection of GFP mRNA‐loaded LNPs into 4T1 tumors (n = 3). (P) CCK8 assay of 4T1 cells transfected with COM‐LNPs or LPC‐LNPs with indicated concentration of siNC for 24 h (n = 4). (Q) Flow cytometry analysis of living cells (Zombie^low^) in splenic cells treated with DiI‐labeled LNPs encapsulating GFP mRNA for 24 h (n = 3). Data are presented as mean ± SD or Sem. Statistical significance was analyzed by the two‐way ANOVA, one‐way ANOVA with Tukey's multiple comparison, and unpaired Student's *t*‐test. *p < 0.05, **p < 0.01, ***p < 0.001.

To purposefully optimize intracellular delivery specifically toward cancer cells while evading the mononuclear phagocyte system, we incorporated 16:0 LPC, the main substrate of Lands’ cycle which was highly active in cancer cells, [24, 25] into a standardized LNP formulation (Figure 1A). Besides, two additional candidate lipids, PAF C16, an analogue of 16:0 LPC, and 16:0 Diether PC, which was used for preparing LNP and artificial lipid membrane, were also individually incorporated (Figure 1A). These specific lipid candidates differed in acyl/alkyl chain composition and linkage chemistry, which may influence both the efficiency of cellular internalization and the subsequent endosomal escape and translation of the encapsulated RNA payload. To systematically evaluate the delivery efficacy of these metabolic modifications, a benchmark control formulation was generated utilizing the classical four‐component system comprising the ionizable lipid SM‐102, DSPC, cholesterol, and DMG‐PEG2000. This standard baseline was designated the commercial‐like formulation, or COM‐LNP.

Initial high‐throughput screening was executed utilizing a rapid vortex‐mixing synthesis protocol to efficiently encapsulate luciferase reporter mRNA. The generated arrays of modified nanoparticles were subsequently evaluated in vitro using 4T1 murine triple‐negative breast cancer (TNBC) cells. Remarkably, following 12 h of incubation, LNPs modified with 16:0 LPC with a 14 molar ratio yielded a remarkable threefold enhancement in luciferase expression compared to formulations with either of the other two candidate lipids or COM‐LNPs (Figure 1B). This superior transfection capability was not a transient artifact; it was consistently maintained during extended incubation assessments spanning 24 and 48 h, highlighting the sustained biochemical stability and enduring translational activity of the delivered payload (Figure 1B). Based on these systematic screens, the formulation containing 16:0 LPC with a 14 molar ratio was selected as the optimal lipid composition (Figure 1C), designated LPC‐LNP. COM‐LNPs and LPC‐LNPs were prepared by microfluidic mixing, with lipids dissolved in the organic phase and RNA in the aqueous phase (Figure 1D).

To evaluate whether the massive incorporation of 16:0 LPC altered the structural integrity or thermodynamic stability of the nanoparticle system, the COM‐LNPs and LPC‐LNPs were precisely synthesized utilizing advanced microfluidic mixing protocols and then characterized. Dynamic light scattering (DLS) analysis revealed that LPC‐LNPs maintained a highly stable hydrodynamic diameter tightly analogous to COM‐LNPs (≈150 nm) and showed a narrow size distribution (Figure 1E), ensuring their optimal suitability for in vivo administration. Transmission electron microscopy (TEM) further confirmed that LPC‐LNP constructs displayed a uniform, monodisperse spherical morphology entirely free from structural aggregation (Figure 1F). Surface charge assessments yielded comparable, slightly negative zeta potentials nearing −2.5 mV for both the standard and modified formulations (Figure 1G). Importantly, 16:0 LPC incorporation did not compromise nucleic acid loading, achieving an RNA encapsulation efficiency nearly 100% (Figure 1H). Overall, the addition of 16:0 LPC did not adversely affect the essential physical and chemical properties of the LNPs, thereby providing a stable formulation for delivery studies.

Next, we investigated the in vitro delivery performance of LPC‐LNPs utilizing Cy5‐fluorophore‐labeled siRNA, revealing that the LPC‐LNP platform mediated exceptionally rapid and highly efficient cellular internalization kinetics. Flow cytometry profiling over a continuous 24‐hour monitoring period showed that the mean fluorescence intensity (MFI) of Cy5 in 4T1 cells treated with LPC‐LNPs was approximately fourfold greater than that of the cells exposed to the COM‐LNPs baseline (MFI: 22089 ± 1147.2 vs. 5203 ±89.7) (Figure 1I). These results indicated that incorporation of 16:0 LPC enhanced nanoparticle‐cell interactions and increased the amount of siRNA delivered into tumor cells. To elucidate the exact molecular mechanics driving this enhanced cellular uptake of LPC‐LNPs, cancer cells were pretreated with a highly selective panel of pharmacological endocytosis inhibitors, including amiloride (macropinocytosis inhibitor), [33] chlorpromazine (CPZ; clathrin‐mediated endocytosis inhibitor), [34] genistein (caveolin‐mediated endocytosis inhibitor), [35] and dynasore (global dynamin inhibitor that affects both clathrin‐ and caveolin‐dependent internalization) [36]. While amiloride exhibited negligible effects on LPC‐LNP internalization, both chlorpromazine and genistein induced modest but statistically significant reductions (decreasing the uptake from 99.8% to 84.8% and to 67.4%, respectively) (Figure 1J). Conversely, cellular treatment with dynasore precipitated a drastic, immediate decrease in particle internalization down to 51.6% of the untreated baseline, strongly indicating that the LPC‐LNPs heavily exploit both clathrin‐ and caveolin‐dependent endocytic pathways for initial cellular entry (Figure 1J). Furthermore, inhibiting 4T1 cell Lands‘ cycle by silencing LPCAT enzymes (Figure S2A–D) impaired the cellular uptake of LPC‐LNPs (Figure 1K), supporting our hypothesis that active Lands‘ cycle in cancer cells might contribute to enhanced uptake of LPC‐LNPs. The downstream translational efficacy, strictly indicative of successful endosomal escape and cytosolic payload release, was confirmed utilizing a green fluorescent protein (GFP) mRNA payload. LPC‐LNPs elicited a robust, dose‐dependent expression profile that vastly eclipsed COM‐LNPs at every evaluated time point, achieving a staggering 19‐fold maximum enhancement in fluorescence intensity at 96 h post‐transfection (Figure 1L,M). These results strongly suggest that LPC‐LNPs not only improve delivery efficiency but also support sustained intracellular translation of RNA.

A major physiological obstacle in nanomedicine for solid tumors is the rapid and non‐specific uptake and clearance of circulating nanoparticles by the mononuclear phagocyte system, primarily mediated by tissue‐resident macrophages and circulating monocytes [37, 38, 39]. To extensively profile the systemic targeting characteristics of LPC‐LNPs, its functional distribution was assessed within the highly diverse immune cell subsets of murine splenocytes. Compared with COM‐LNPs, the LPC‐LNP formulation demonstrated a substantially diminished propensity to transfect immune populations, recording significantly lower uptake signals across multiple immune cell subsets, including T cells, B cells, NK cells, and dendritic cells (Figure 1N; Figure S3A,B). This vital stealth trend was rigorously conserved within the highly suppressive tumor microenvironment following the direct intratumoral injection of GFP mRNA‐loaded LNPs (Figure 1O; Figure S4A,B). The COM‐LNPs baseline exhibited massive, non‐specific accumulation within tumor‐associated macrophages (TAMs) and monocytes. Conversely, the 16:0 LPC modification actively evaded monocytes and TAMs‐mediated clearance. Simultaneously, the tumor cell‐specific transfection efficiency soared from 9.5% in the COM‐LNP group to 16.0% in the LPC‐LNP group, representing a highly significant ∼1.7‐fold localized enhancement. Besides, LPC‐LNPs displayed little transfection in CD4^+^ T cells, CD8^+^ T cells, B cells, NK, cDC1, and endothelial cells. Overall, the rational incorporation of 16:0 LPC not only amplifies transfection at the cellular level but also provides exceptional cancer cell specificity while completely bypassing the critical immune cells required for ultimate tumor eradication. Besides, LPC‐LNPs showed no additional cellular toxicity in 4T1 cells and splenic immune cells compared with COM‐LNPs (Figure 1P,Q). Together, the findings establish this formulation as a highly potent and translational platform for tumor‐targeted RNA delivery.

### 2.2 LPC‐LNP‐Delivered Cd28 siRNA Selectively Knocks Down Cancer Cell CD28

**FIGURE 2:**
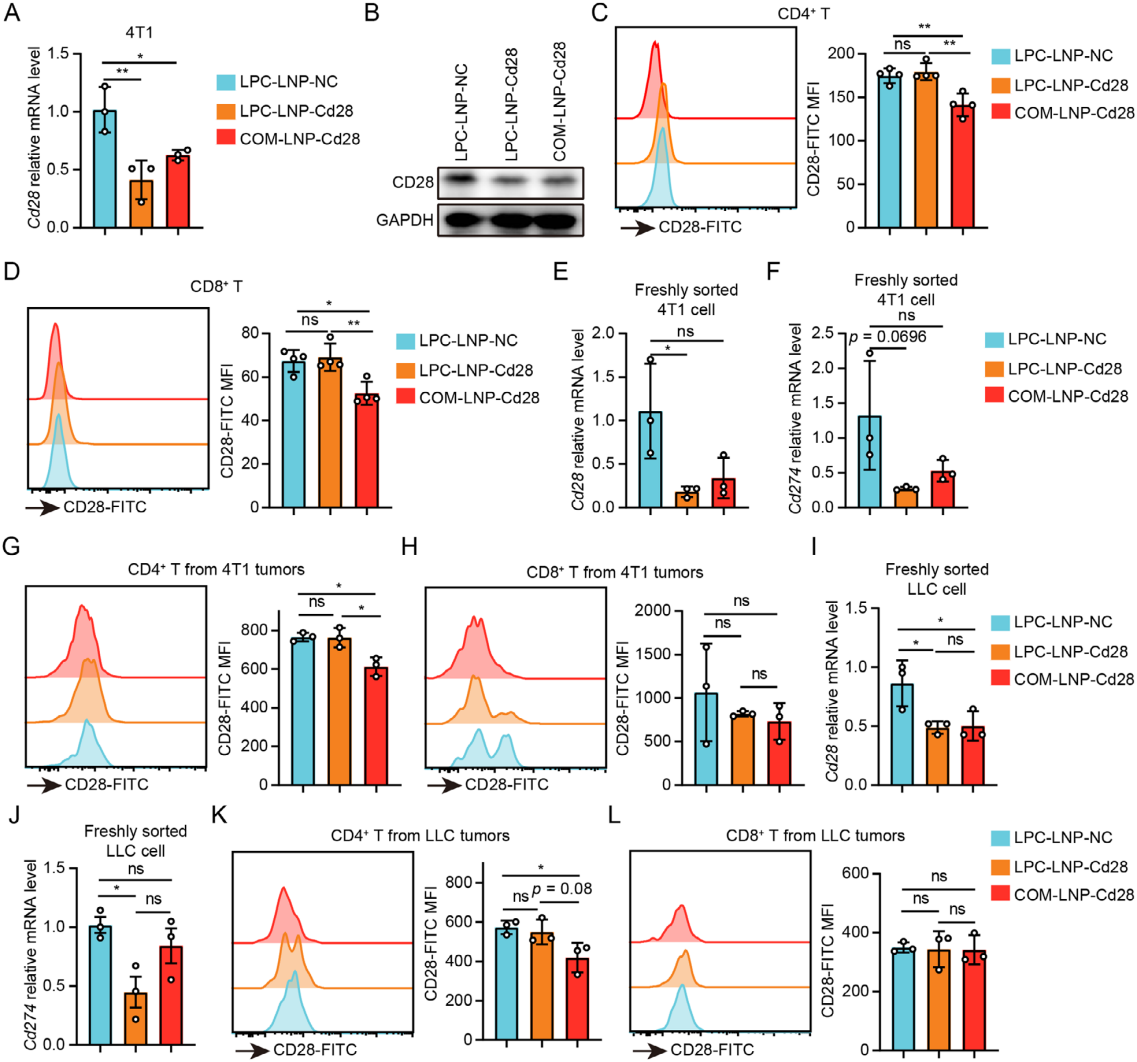
*Cd28* siRNA delivery by LPC‐LNPs selectively knocks down cancer cell CD28. (A, B) Q‐PCR (A) and Western blot (B) analysis of *Cd28* silence efficacy in 4T1 cells delivered si*Cd28* by LPC‐LNPs or COM‐LNPs for 60 h in 4T1 cells (n = 3). (C, D) CD4^+^ T cell (C) and CD8^+^ T cell (D) membrane CD28 expression level analyzed by flow cytometry after splenocytes were treated with LPC‐LNP‐NC, LPC‐LNP‐Cd28, or COM‐LNP‐Cd28 for 24 h (n = 4). (E, F) *Cd28* (E) and *Cd274* (F) mRNA level analyzed by Q‐PCR of freshly sorted 4T1 cells from 4T1 tumors receiving 3 doses of LPC‐LNP‐NC, LPC‐LNP‐Cd28, or COM‐LNP‐Cd28 intratumorally injection 10 days after tumor inoculation (n = 3). (G, H) CD4^+^ T cell (G) and CD8^+^ T cell (H) membrane CD28 level analyzed by flow cytometry from 4T1 tumors receiving 3 doses of LPC‐LNP‐NC, LPC‐LNP‐Cd28, or COM‐LNP‐Cd28 intratumoral injection 10 days after tumor inoculation (n = 3). (I, J) *Cd28* (I) and *Cd274* (J) mRNA level analyzed by Q‐PCR of freshly sorted LLC cells from LLC tumors receiving 3 doses of LPC‐LNP‐NC, LPC‐LNP‐Cd28, or COM‐LNP‐Cd28 intratumorally injection 10 days after tumor inoculation (n = 3). (K, L) CD4^+^ T cell (K) and CD8^+^ T cell (L) membrane CD28 level analyzed by flow cytometry from LLC tumors receiving 3 doses of LPC‐LNP‐NC, LPC‐LNP‐Cd28, or COM‐LNP‐Cd28 intratumorally injection 10 days after tumor inoculation (n = 3). Data are presented as mean ± Sem. Statistical significance was analyzed by one‐way ANOVA analysis with Tukey's multiple comparison, *p < 0.05, **p < 0.01.

Building directly upon our previous report that cancer cell intracellular CD28 mediates cancer immune escape via the post‐transcriptional stabilization of *CD274* mRNA and inhibiting cancer cell CD28 can overcome anti‐PD‐1 resistance, [15] the capacity of the newly developed, cancer cell‐targeted LPC‐LNP platform to facilitate cancer‐selective *Cd28* knockdown was evaluated. Firstly, we confirmed that the application of LPC‐LNPs encapsulating *Cd28* siRNA (LPC‐LNP‐Cd28) elicited a profound, rapid reduction in both *Cd28* mRNA transcripts and overall intracellular CD28 protein levels in 4T1 cells, achieving a silencing efficacy fundamentally comparable to highly transfective COM‐LNP‐Cd28 (Figure 2A,B). When murine splenocytes were treated with LPC‐LNP‐Cd28 or COM‐LNP‐Cd28 for 24 h, the non‐specific, universally internalizing COM‐LNP‐Cd28 abrogated membrane CD28 expression across both the CD4^+^ and CD8^+^ T cell populations, but the LPC‐LNP‐Cd28 left T cell CD28 surface densities entirely unperturbed (Figure 2C,D). This explicitly mirrors the established cancer cell specificity inherent to the 16:0 LPC modification and confirms the safety of the LPC‐LNP platform regarding lymphoid viability.

We then tested the efficiency and specificity of LPC‐LNP‐Cd28 in vivo. After the mice harboring mature orthotopic 4T1 breast tumors were subjected to 3 sequential intratumoral injections of either a nonsense negative control sequence (LPC‐LNP‐NC), the targeted LPC‐LNP‐Cd28, or the non‐specific COM‐LNP‐Cd28 starting exactly 10 days post‐tumor inoculation, we sorted CD45^−^EpCAM^+^ epithelial tumor cells directly from the excised tumor tissues (Figure S5). Q‐PCR analysis confirmed that both COM‐LNP‐Cd28 and LPC‐LNP‐Cd28 successfully and equally knocked down *Cd28* in the sorted tumor cells (Figure 2E). Besides, COM‐LNP‐Cd28 and LPC‐LNP‐Cd28 decreased *Cd274* mRNA level in the tumor cells (Figure 2F), consistent with our previous discoveries that cancer cell CD28 stabilized *Cd274* mRNA and promoted PD‐L1 expression [15]. As for T cells, COM‐LNP‐Cd28 decreased the membrane CD28 of tumor‐infiltrating CD4^+^ T cells, while LPC‐LNP‐Cd28 showed no effects (Figure 2G). Unlike in vitro results, both COM‐LNP‐Cd28 and LPC‐LNP‐Cd28 rarely disturbed CD8^+^ T cell CD28 expression (Figure 2H), probably caused by replacement CD8^+^ T cells entering the tumor microenvironment from sites outside the tumor [40]. Similar results were also observed in a murine model bearing subcutaneous Lewis lung cancer (LLC). COM‐LNP‐Cd28 and LPC‐LNP‐Cd28 reduced *Cd28* and *Cd274* mRNA levels in freshly sorted LLC cells (Figure 2I,J). LPC‐LNP‐Cd28 retained CD4^+^ T cell and CD8^+^ T cell CD28 expression, while COM‐LNP‐Cd28 impaired CD4^+^ T cell CD28 expression (Figure 2K,L). Collectively, these data demonstrate that LPC‐LNP‐Cd28 effectively silences cancer cell CD28 with tumor‐selective targeting without interfering with T cell CD28 expression.

### 2.3 Intratumoral Injection of LPC‐LNP‐Cd28 Inhibits Tumor Growth

**FIGURE 3:**
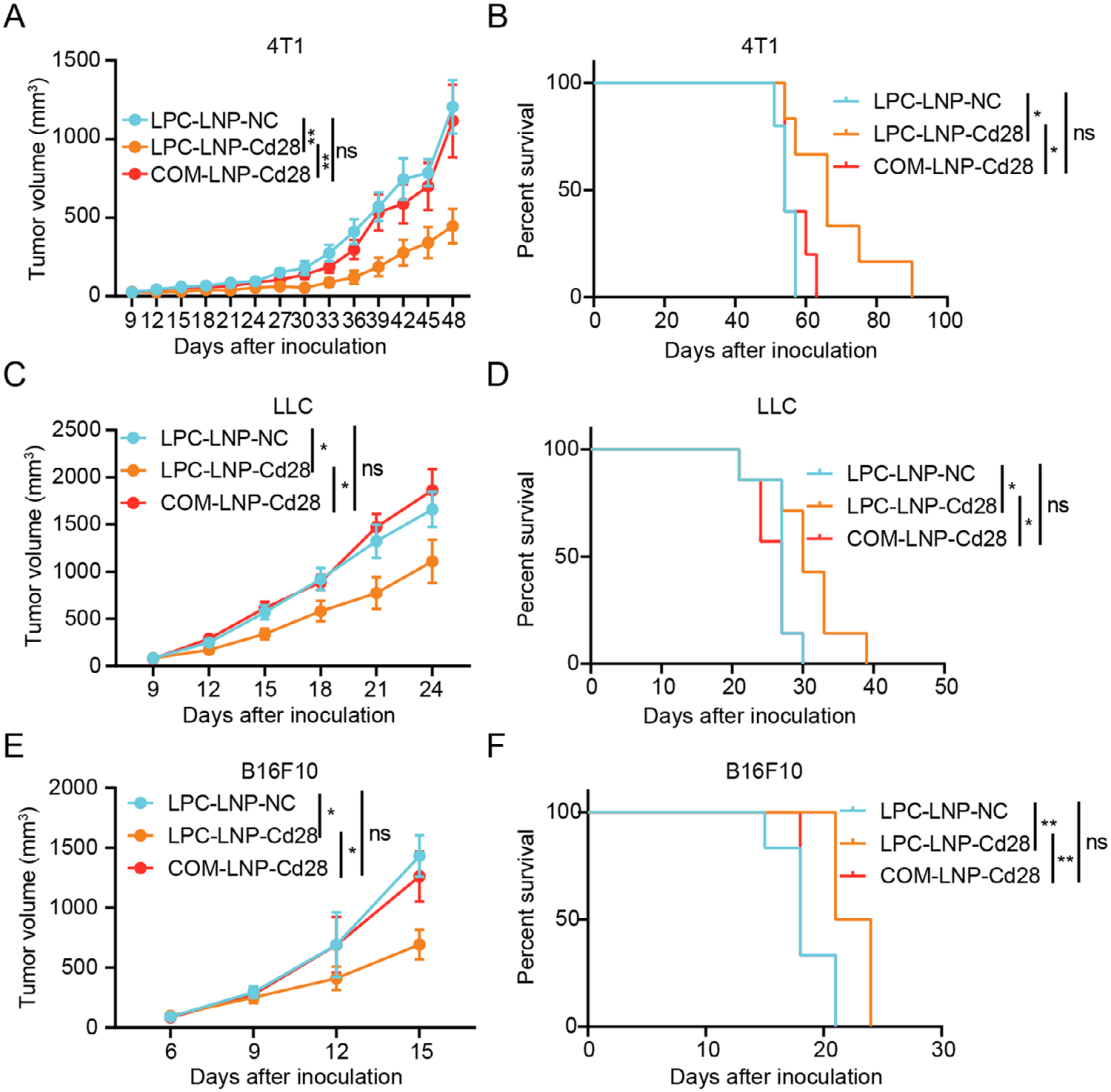
Intratumoral *Cd28* siRNA delivery by LPC‐LNPs inhibits tumor growth. (A, B) Tumor growth (A) and tumor‐bearing mice survival (B) of 4T1 tumors intratumorally injected with indicated LNP containing 20 µg siRNA at day 10,12 and 14 after tumor inoculation (n = 5 or 6). (C, D) Tumor growth (C) and tumor‐bearing mice survival (D) of LLC tumors intratumorally injected with indicated LNP containing 20 µg siRNA at day 10,12 and 14 after tumor inoculation (n = 7). (E, F) Tumor growth (E) and tumor‐bearing mice survival (F) of B16F10 tumors intratumorally injected with indicated LNP containing 20 µg siRNA at day 10,12 and 14 after tumor inoculation (n = 6). Data are presented as mean ± Sem. Statistical significance was analyzed by two‐way ANOVA and log‐rank test, *p < 0.05, **p < 0.01.

With the biochemical specificity and functional safety of the LPC‐LNP‐Cd28 definitively established to selectively knock down cancer cell CD28, we further explored the antitumor effects of LPC‐LNP‐Cd28 in the murine orthotopic 4T1 TNBC model, which shows resistance to anti‐PD‐1 immunotherapy [41, 42]. LNP containing 20 µg siRNA was intratumorally administered 3 times at day 10, 12, and 14 after tumor inoculation. The highly aggressive tumors treated with unoptimized COM‐LNP‐Cd28 exhibited a similar growth rate compared with the control group receiving LPC‐LNP‐NC treatment. Conversely, the localized administration of the target‐specific LPC‐LNP‐Cd28 significantly repressed tumor growth (Figure 3A). Besides, LPC‐LNP‐Cd28 prolonged the survival of tumor‐bearing mice, which was absent in COM‐LNP‐Cd28‐treated mice (Figure 3B). In the murine LLC subcutaneous tumor model with resistance to anti‐PD‐1 immunotherapy, [43, 44] LPC‐LNP‐Cd28 also inhibited tumor growth and prolonged the survival of tumor‐bearing mice, but COM‐LNP‐Cd28 failed to exert any measurable control over tumor growth (Figure 3C,D). Furthermore, the LPC‐LNP‐Cd28 administration powerfully suppressed tumor growth in the B16F10 melanoma tumor model, which was a classic “cold” tumor resisting anti‐PD‐1 therapy, [45] however, COM‐LNP‐Cd28 proved entirely useless (Figure 3E,F). Thus, LPC‐LNP‐Cd28 intratumoral injection showed potent antitumor ability in multiple mouse tumor models, while COM‐LNP‐Cd28 failed probably owing to off‐target effects on T cells. Furthermore, these results suggest the therapeutic potential encapsulated in specifically and exclusively inhibiting cancer cell CD28 without harming the host's adaptive immune infrastructure.

### 2.4 Intratumoral Injection of LPC‐LNP‐Cd28 Activates CD8+ T Cell Antitumor Immunity

**FIGURE 4:**
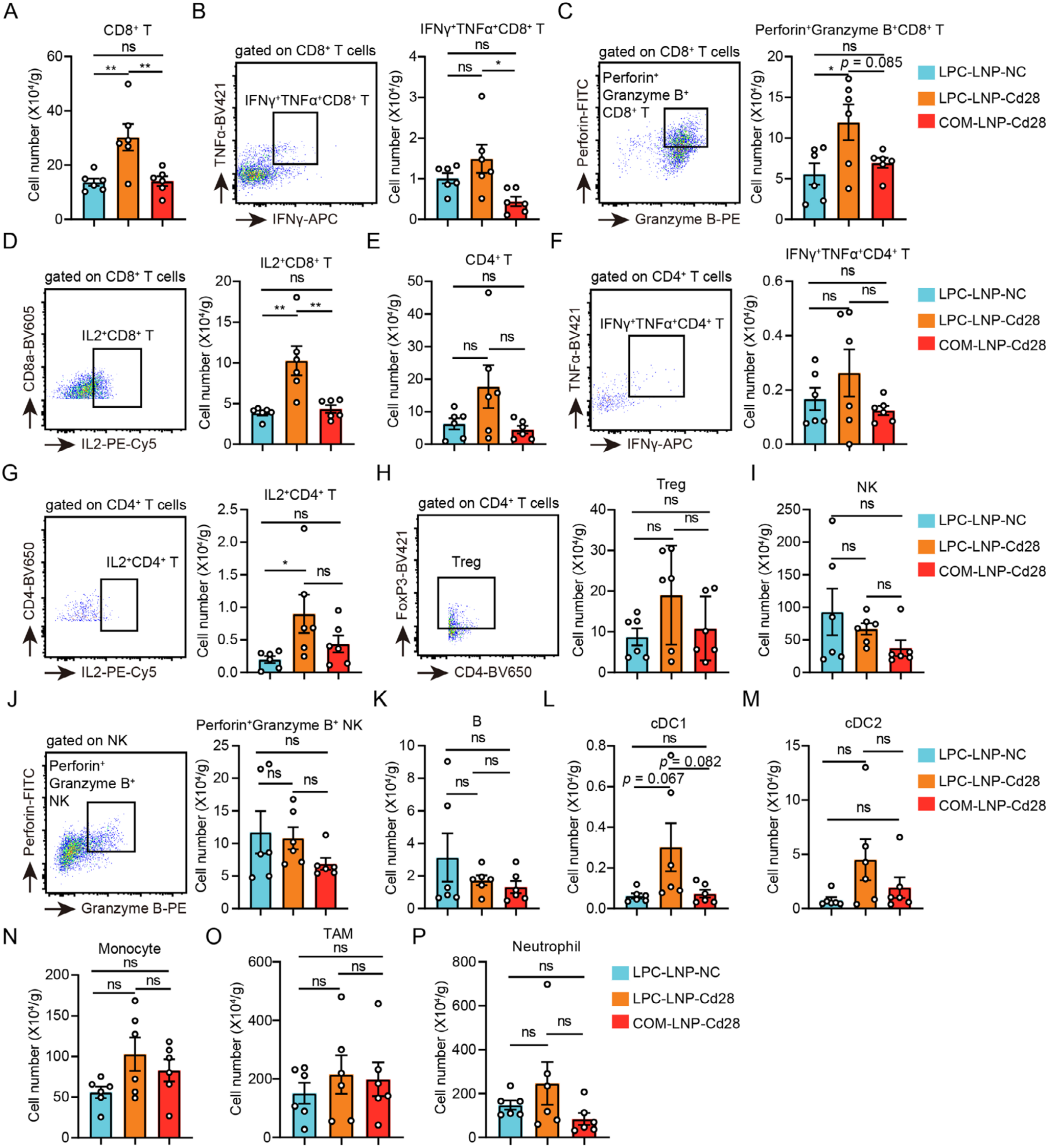
Intratumoral *Cd28* siRNA delivery by LPC‐LNPs activates CD8^+^ T cell antitumor immune response. (A–D) CD8^+^ T cells (A), IFNγ^+^TNFα^+^CD8^+^ T cells (B), Perforin^+^Granzyme B^+^CD8^+^ T cells (C) and IL2^+^CD8^+^ T cells (D) analyzed by flow cytometry in 4T1 tumors intratumorally injected with indicated LNP containing 20 µg siRNA for 3 times (n = 6). (E‐H) CD4^+^ T cells (E), IFNγ^+^TNFα^+^CD4^+^ T cells (F), IL2^+^CD4^+^ T cells (G), and Tregs (H) analyzed by flow cytometry in 4T1 tumors (n = 6). (I, J) NK (I) and Perforin^+^Granzyme B^+^NK (J) analyzed by flow cytometry in 4T1 tumors (n = 6). (K‐P) B cells (K), cDC1 (L), cDC2 (M), Monocyte (N), TAM (O), and Neutrophil (P) analyzed by flow cytometry in 4T1 tumors (n = 6). Data are presented as mean ± Sem. Statistical significance was analyzed by one‐way ANOVA analysis with Tukey's multiple comparison, *p < 0.05, **p < 0.01.

We next aimed to uncover how LPC‐LNP‐Cd28 remodels the immunosuppressive TME and induces antitumor immunity to suppress tumor growth (Figure S6A, B). Flow cytometry analysis showed that treatment with LPC‐LNP‐Cd28 triggered a massive, highly significant expansion in the absolute quantification of infiltrating CD8^+^ T cells within the 4T1 and LLC tumors, while an immunological enhancement was entirely lacking in both the COM‐LNP‐Cd28 and negative control (Figure 4A, Figure S7A and S8A). Besides, CD8^+^ T cell activation, as indicated by IFNγ and TNFα expression, was also enhanced by LPC‐LNP‐Cd28 instead of COM‐LNP‐Cd28 (Figure 4B; Figures S7B and S8B). Perforin^+^Granzyme B^+^CD8^+^ T cells were elevated after LPC‐LNP‐Cd28 treatment, suggesting superior cytotoxicity to cancer cells, which phenomenon was absent in tumors treated with COM‐LNP‐Cd28 (Figure 4C; Figures S7C and S8C). CD8^+^ T cells from tumors treated with LPC‐LNP‐Cd28 also expressed more IL2 than those treated with LPC‐LNP‐NC or COM‐LNP‐Cd28 (Figure 4D; Figures S7D andS8D). Therefore, intratumoral injection of LPC‐LNP‐Cd28 enhances CD8^+^ T cell infiltration, activation, and cytotoxicity, along with the potent suppression of tumor growth.

While the localized delivery of LPC‐LNP‐Cd28 tended to consistently increase infiltration numbers of CD4^+^ T cells in TME, the numerical shift did not cross statistical significance bounds (Figure 4E; Figures S7E and S8E). However, the functional quality of the helper T cell pool improved dramatically. This was evidenced by potent biological trends toward elevated IFNγ, TNFα, and IL2 production among the helper subsets (Figure 4F,G; Figures S7F,G and S8F,G). Although COM‐LNP‐Cd28 decreased cancer cell *Cd28* and *Cd274* as mentioned before (Figure 2E,F,I,J), COM‐LNP‐Cd28 knocked down *Cd28* in CD4^+^ T cells in the meantime (Figure 2G,K). The reduced CD28 in CD4^+^ T cells might account for the failure to activate CD8^+^ T cells in tumors treated with COM‐LNP‐Cd28, since CD28‐mediated stimulatory signaling in CD4^+^ T cells is critical for CD8^+^ T cell antitumor immune response [46]. As for regulatory T cells (Tregs), neither LPC‐LNP‐Cd28 nor COM‐LNP‐Cd28 altered Treg in 4T1 tumors (Figure 4H; Figure S7H). In contrast, COM‐LNP‐Cd28 treatment increased Treg abundance in LLC tumors (Figure S8H), which might further suppress CD8^+^ T cells. Although LPC‐LNP‐Cd28 and COM‐LNP‐Cd28 tended to reduce NK cell abundance and function in 4T1 tumors, the phenomenon was absent in LLC tumors (Figure 4I,J; Figures S7I,J and S8I,J). A similar change of B cells was observed as with NK cells (Figure 4K; Figures S7K and S8K).

Deep, high‐resolution profiling of the myeloid cellular compartments yielded complementary, highly essential insights into the mechanism of therapeutic success. Selective CD28 knockdown by LPC‐LNP‐Cd28 orchestrated a profound localized accumulation of conventional type 1 dendritic cells (cDC1s) in TME (Figure 4L; Figures S7L and S8L), which was consistent with enhanced CD8^+^ T cell function, since cDC1‐mediated cross‐presentation is indispensable for CD8^+^ T cell activation [47]. Besides, conventional type 2 dendritic cells (cDC2) similarly exhibited strong upward migratory trends in LLC tumors following targeted treatment (Figure 4M; Figures S7M and S8M). In 4T1 tumors, neither LPC‐LNP‐Cd28 nor COM‐LNP‐Cd28 altered the number of monocytes, tumor‐associated macrophages (TAMs), or immunosuppressive neutrophils (Figure 4N–P; Figure S7N–P). In LLC tumors, COM‐LNP‐Cd28 is intended to increase the abundance of monocytes, TAMs, and neutrophils (Figure S8N–P), especially neutrophils. The increase of Tregs and myeloid cells in LLC tumors after COM‐LNP‐Cd28 administration was consistent with previous research about the connection between Tregs and myeloid cells, revealing that Tregs recruited myeloid cells to TME while myeloid cells also recruited Tregs in return [48].

Together, the results suggest that intratumoral injection of LPC‐LNP‐Cd28 increases CD8^+^ T cell infiltration and enhances CD8^+^ T cell antitumor immunity in TME, leading to tumor control. COM‐LNP‐Cd28 fails to render CD8^+^ T cell antitumor immunity, probably caused by CD28 loss in CD4^+^ T cells.

### 2.5 Intratumoral Injection of LPC‐LNP‐Cd28 Overcomes Anti‐PD‐1 Immunotherapy Resistance

**FIGURE 5:**
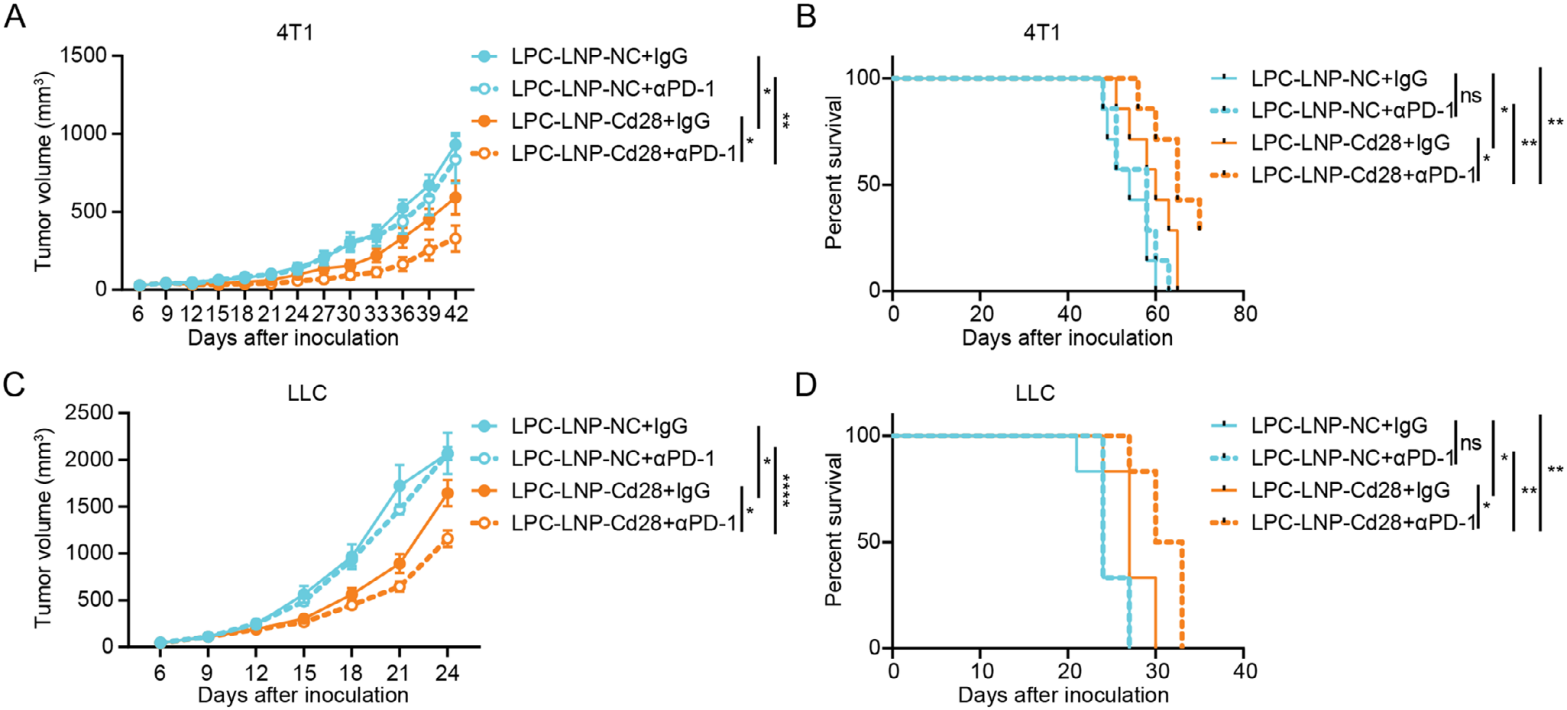
Intratumoral *Cd28* siRNA delivery by LPC‐LNPs overcomes anti‐PD‐1 resistance. (A, B) Tumor growth (A) and tumor‐bearing mice survival (B) of 4T1 tumors intratumorally injected with indicated LNPs containing 20 µg siRNA at day 10, 12, 14, and intraperitoneally injected with 200 µg IgG/anti‐PD‐1 antibody at day 9, 12, 15, 18 after tumor inoculation (n = 7). (C, D) Tumor growth (C) and tumor‐bearing mice survival (D) of LLC tumors intratumorally injected with indicated LNPs and IgG/anti‐PD‐1 antibody treatment (n = 6). Data are presented as mean ± Sem. Statistical significance was analyzed by two‐way ANOVA and log‐rank test, *p < 0.05, **p < 0.01, ***p < 0.001, ****p < 0.0001.

The clinical efficacy of systemically administered anti‐PD‐1 antibody (αPD‐1) relies almost entirely upon the presence of a pre‐existing, highly tumor‐reactive T cell infiltrate that is temporarily incapacitated by localized checkpoint ligation [4]. Given that LPC‐LNP‐Cd28 treatment activated CD8^+^ T cells in TME, the combination of locally administered LPC‐LNP‐Cd28 with intraperitoneal αPD‐1 therapy was evaluated in the “immunologically cold” orthotopic 4T1 carcinoma model. As widely anticipated, systemic αPD‐1 monotherapy exhibited total functional failure, demonstrating absolutely no capability to arrest aggressive tumor expansion. Single‐agent LPC‐LNP‐Cd28 intratumoral injection inhibited tumor growth as mentioned before. Strikingly, combining LPC‐LNP‐Cd28 and αPD‐1 triggered an immense synergistic antitumoral effect compared with single‐agent treatments (Figure 5A). Concomitantly, the combinatorial regimen dramatically enhanced the overall survival rates compared to LPC‐LNP‐Cd28, while αPD‐1 alone had no such effect (Figure 5B). Furthermore, the potent antitumor effect was also confirmed in the mouse subcutaneous LLC tumor model treated with the combinatorial regimen. Similarly, LPC‐LNP‐Cd28 rather than αPD‐1 treatment inhibited tumor growth, and LPC‐LNP‐Cd28 plus αPD‐1 treatment more potently suppressed tumor growth (Figure 5C). Combining LPC‐LNP‐Cd28 and αPD‐1 showed the same survival benefits in LLC tumors compared with 4T1 tumors (Figure 5D). These data indicate that intratumoral injection of LPC‐LNP‐Cd28 not only suppresses tumor growth but also overcomes the resistance of 4T1 and LLC tumors to αPD‐1 therapy, achieving more pronounced antitumor effects.

### 2.6 Enhanced Antitumor Immunity In Vivo After Combined LPC‐LNP‐Cd28/Anti‐PD‐1 Therapy

**FIGURE 6:**
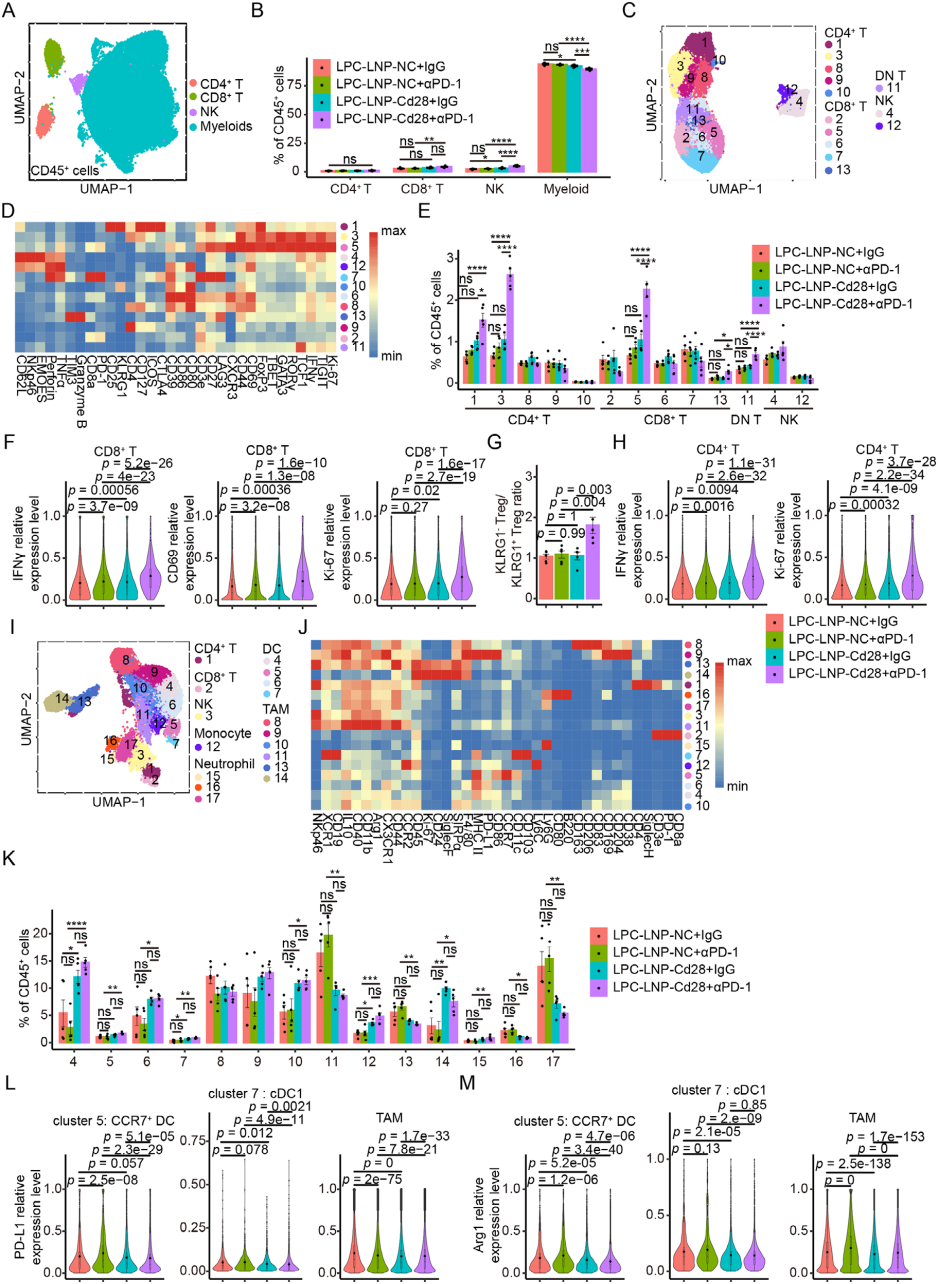
LPC‐LNP‐Cd28 and anti‐PD‐1 combined treatment in 4T1 tumors enhanced antitumor immunity. (A) UMAP projection of CD45^+^ cells colored by major clusters from 4T1 tumors receiving LPC‐LNP‐Cd28 and αPD‐1 combined treatment, highlighting lymphoid cells by CyTOF from the lymphoid‐centric panel. (B) Proportion of lymphoid cluster in total CD45^+^ cells from lymphoid‐centric panel (n = 5). (C) UMAP projection of lymphoid cells colored by PhenoGraph clusters. (D) Normalized marker expression of each lymphoid cluster. (E) Proportion of each lymphoid cluster in CD45^+^ cells (n = 5). (F) Violin plot showing IFNγ, CD69, and Ki‐67 expression in CD8^+^ T cells. (G) The ratio of KLRG1^−^ and KLRG1^+^ Tregs in different groups (n = 5). (H) Violin plot showing IFNγ and Ki‐67 expression in CD4^+^ T cells. (I) UMAP projection of CD45^+^ cells colored by PhenoGraph clusters from 4T1 tumors receiving LPC‐LNP‐Cd28 and αPD‐1 combined treatment by CyTOF from myeloid‐centric panel. (J) Normalized marker expression of each cluster from myeloid‐centric panel. (K) Proportion of each lymphoid cluster in CD45^+^ cells (n = 5). (L) Violin plot showing PD‐L1 expression in cluster 5, cluster 7, and TAM. (M) Violin plot showing Arg1 expression in cluster 5, cluster 7, and TAM. Data are presented as mean ± Sem. Statistical significance was analyzed by one‐way ANOVA analysis with Tukey's multiple comparison or Wilcoxon rank‐sum test, *p < 0.05, **p < 0.01, ***p < 0.001, ****p < 0.0001.

To debarcode TME remodeling following the combinatorial application of LPC‐LNP‐Cd28 and systemic αPD‐1 therapy, we performed single‐cell mass cytometry (cytometry by time‐of‐flight, CyTOF) to further analyze the immunophenotyping of 4T1 tumors after the treatment using a lymphoid‐centric and a myeloid‐centric panel. Firstly, we analyzed the lymphoid compartment of the TME with the lymphoid‐centric panel containing 38 antibodies covering classic cell markers, function, and status‐associated molecules (Table S1). We performed unsupervised clustering analysis using the Louvain clustering‐based Phenograph algorithm [49] and reduced the dimensionality of the data for visualization by uniform manifold approximation and projection (UMAP) [50]. We obtained 25 different clusters among CD45^+^ immune cells (Figure S9A), which were assigned to CD4^+^ T cells, CD8^+^ T cells, NKs, and myeloid cells (Figure 6A; Figure S9B). Combined treatment significantly promoted CD8^+^ T cells and NK cells accumulation, while the percentage of myeloid cells decreased in the LPC‐LNP‐Cd28 and combined group (Figure 6B). As expected, αPD‐1 treatment alone failed to stimulate or recruit CD8^+^ T cells into the tumor tissue (Figure 6B), offering a molecular explanation for their absolute clinical failure in monotherapy settings.

To deeply analyze the lymphoid cell architecture, we performed second‐round clustering and UMAP analysis on lymphoid cells, including CD4^+^ T cells, CD8^+^ T cells, and NK cells. We got 13 distinct clusters with featured molecule expression profiles (Figure 6C; Figure S10). Among the 13 clusters, 5 belonged to CD4^+^ T cell (cluster 1, 3, 8, 9, 10), 5 belonged to CD8^+^ T cell (cluster 2, 5, 6, 7, 13), 1 double‐negative T cell (DN T) cluster (cluster 11) and 2 belonged to NK cell (cluster 4, 12) (Figure 6C; Figure S9). In CD8^+^ T cells, cluster 5 expressed high level IFNγ and Ki‐67, which represented activated CD8^+^ T cells, and was mostly enriched after LPC‐LNP‐Cd28 and αPD‐1 combined treatment (Figure 6D,E). Besides, cluster 13 with the highest Granzyme B expression representing cytotoxicity CD8^+^ T cells increased significantly after combined treatment (Figure 6D,E). Furthermore, CD8^+^ T cells from the LPC‐LNP‐Cd28 single treatment group increased IFNγ, CD69, and Ki‐67 expression, which were further increased by combined treatment (Figure 6F). As for CD4^+^ T cells, Treg cells (cluster 1 and 3) were elevated after combined treatment (Figure 6D,E). KLRG1^+^ Tregs represented a terminally differentiated Treg subset with higher immunosuppressive activity derived from KLRG1^−^ Tregs [51]. The relative ratio between KLRG1^−^ Tregs (cluster 3) and KLRG1^+^ Tregs (cluster 1) increased after combined treatment (Figure 6G), indicating that the differentiation of KLRG1^−^ Tregs was hindered. Besides, IFNγ and Ki‐67 expression in CD4^+^ T cells increased after combined treatment (Figure 6H), indicative of enhanced activation status. DN T (cluster 11) also increased after combined treatment (Figure 6E).

We then profiled the myeloid compartment using the myeloid‐centric panel containing 37 antibodies (Table S2). Phenograph and UMAP analysis identified 17 clusters after manual integration (Figure 6I; Figure S11). We then focused on 14 myeloid clusters including DCs, TAMs, monocytes, and neutrophils. Cluster 4 and cluster 6 belonged to cDC2, which increased after combined treatment, and cluster 4 also increased after the LPC‐LNP‐Cd28 single treatment (Figure 6J,K). More importantly, cluster 7, which was featured with CD103 and XCR1 expression resembling cDC1, was elevated after the LPC‐LNP‐Cd28 single treatment and combined treatment (Figure 6J,K), consistent with enhanced CD8^+^ T cell antitumor immune response. Besides, CCR7^+^ DC (cluster 5), which had been reported to augment CD8^+^ T cell cytolytic activity following anti‐PD‐L1 therapy, [52] increased after combined treatment (Figure 6J,K). Furthermore, cDC1 and CCR7^+^ DC from LPC‐LNP‐Cd28 and combined treatment group exhibited reduced inhibitory molecules including PD‐L1 and Arg1 (Figure 6L,M). For myeloid cells with immunosuppressive features, TAM (clusters 11 and 13) and neutrophils (clusters 16 and 17) expressing IL10 and Arg1 decreased after combined treatment (Figure 6L,M). Meanwhile, PD‐L1 and Arg1 expression in TAM decreased after LPC‐LNP‐Cd28 and the combined treatment (Figure 6L,M). Monocyte (cluster 12) was increased after single‐agent LPC‐LNP‐Cd28 or combined treatment (Figure 6J,K).

Collectively, our CyTOF analysis indicates that combining LPC‐LNP‐Cd28 and αPD‐1 treatment effectively activates cDC1, CCR7^+^ DC and CD8^+^ T cells in TME, which are the main drivers and contributors of antitumor immunity. Also, combined treatment decreases immunosuppressive components in TME, including TAM and neutrophils expressing IL10.

## 3 Discussion

Our findings reveal that specifically calibrated 16:0 LPC‐modified SM‐102‐based LNP (LPC‐LNP) possesses a highly unique, intrinsic capacity to selectively target tumor cells when adjusted at specific formulation without affecting immune cells, especially T cells in TME. This specific formulation, when applied to encapsulate and deliver *Cd28* siRNA, knocked down tumor cell CD28 efficiently while retaining T cell CD28 expression, which leads to the TME remodeling with activated CD8^+^ T cells, consequently inhibiting tumor growth and overcoming anti‐PD‐1 resistance. The fundamental conceptual logic driving the successful development of the LPC‐LNP platform focuses on the specific targeting of cancer cells to overcome tumor‐intrinsic resistance for improving cancer immunotherapy efficacy [53]. Although accumulating researches dedicated to developing organ‐specific LNP [22, 54] and immune cell‐specific LNP [19, 20, 21, 55, 56], much more attention is needed to pay to developing cancer cell‐specific LNP. Besides, most cell‐specific LNPs required the modification of a cell‐specific antibody for targeted delivery [57]. Our LPC‐LNPs achieved the capacity to target tumor cells for the first time to our knowledge via 16:0 LPC rather than antibody modification, which reduced the complexity and expense of LNP synthesis. LPC‐LNPs could be utilized to deliver mRNA encoding neoantigens and modulators as well as siRNA targeting molecules to foster antitumor immunity.

The internalization of LNP by target cells usually depended on clathrin‐mediated and caveolae‐mediated endocytosis or macropinocytosis [58, 59]. We found that COM‐LNPs were internalized by cells mainly through clathrin‐mediated and caveolae‐mediated endocytosis, since inhibition of clathrin‐mediated endocytosis and caveolae‐mediated endocytosis by dynasore [36] or caveolae‐mediated endocytosis by genistein [35] ruined COM‐LNPs cellular uptake, while the macropinocytosis inhibitor amiloride [33] only partially decreased COM‐LNPs cellular uptake. However, LPC‐LNPs cellular uptake seldom relied on macropinocytosis and partially relied on endocytosis, which indicated additional pathways involving LPC‐LNPs cellular uptake. However, further study is still needed to thoroughly exclude macropinocytosis by other classical inhibitors such as ethylisopropylamiloride and brequinar [60]. Additional pathways, such as direct membrane fusion, should also be verified, since soft LNP with a lower Young's modulus predominantly entered cells through membrane fusion [61]. Solid tumors highly upregulate the genomic expression of the LPCAT enzyme family, which converts lysophosphatidylcholine (such as 16:0 LPC) directly into mature PC through targeted fatty acid acylation within the rapid Lands’ cycle [45, 62, 63, 64]. The intentional incorporation of high molar ratios of 16:0 LPC into the structural shell of the nanoparticle effectively masks the entire synthetic construct as a highly desirable, essential metabolic fuel. This might explain the high efficiency and specificity of LPC‐LNPs toward cancer cells. Future research could further explore whether and how LPCAT family in cancer cells affects LPC‐LNPs delivery. After internalization, LNP was transformed to early endosomes and subsequent late endosomes, where nucleic acid payloads must be released into the cytosol before entering lysosomes [65]. Endosomal escape serves as an efficient limiting step for LNP delivery. Whether and how LPC 16:0 influences the endosomal escape process remains to be elucidated.

As a classical co‐stimulatory molecule, CD28 signaling is essential for TCR signaling in both CD4^+^ T cells and CD8^+^ T cells [66, 67]. COM‐LNPs, which also showed effective delivery of *Cd28* siRNA into cancer cells, failed to boost antitumor immunity and inhibit tumor growth probably because it impaired CD28 expression in CD4^+^ T cells. CD28 engagement is also required for follicular helper T‐cell differentiation, [68] which cooperates with B cells to support tertiary lymphoid structure formation and antitumor CD8^+^ T cell immune response [69, 70, 71]. The failure of COM‐LNP‐Cd28 highlighted the necessity of a precise delivery strategy for targets with distinct functions in different cell types. Other potential targets with multifaceted functionality may benefit from our LPC‐LNPs. For example, SIRPα, which suppresses macrophage‐mediated phagocytosis of cancer cells via SIRPα‐CD47 interaction, [72] might promote cancer cell‐CD8^+^ T cell interaction and subsequent cell killing for improving immunotherapy efficacy once *Sipra* mRNA is delivered selectively into cancer cells but not macrophages [73]. The following study will focus on the specificity and safety of LPC‐LNPs in the human body and further test the potential of human *CD28* siRNA delivered by LPC‐LNPs in cancer patient management in the future.

LPC‐LNP‐Cd28 administration increased and activated CD8^+^ T cells and cDC1 in TME, turning “cold” tumors into “hot” tumors and sensitizing tumors to anti‐PD‐1 therapy. CyTOF analysis revealed the immunogenic remodeling after LPC‐LNP‐Cd28 and αPD‐1 combined treatment, with CD8^+^ T cell enrichment and immunosuppressive myeloid cell reduction. Although Tregs increased after combined therapy, the relative ratio between KLRG1^−^ Tregs and KLRG1^+^ Tregs increased. KLRG1 characterized Tregs into two subsets, with KLRG1^+^ Tregs resembling the terminally differentiated Tregs with more potent immunosuppressive function, while KLRG1^−^ Tregs were less differentiated and could transform into KLRG1^+^ Tregs [51, 74, 75]. The combined therapy might inhibit Treg differentiation. PD‐L1 ligation to PD‐1 on Tregs provides critical signals for Tregs induction, differentiation and maintenance [76]. Thus, the combined therapy increased KLRG1^−^ Treg/KLRG1^+^ Treg probably caused by PD‐L1 decreasing in cancer cells by LPC‐LNP‐Cd28. Besides, PD‐L1 expression on myeloid cells also decreased after the combined therapy, which further hindered PD‐1/PD‐L1 signaling for Tregs differentiation. On the other hand, Tregs and immunosuppressive myeloid cells formed a positive feedback loop, with one of which elevated would promote the other [48]. LPC‐LNP‐Cd28 treatment could disrupt this connection. These results provide the mechanistic explanation for the potential synergistic antitumor effect of LPC‐LNP‐Cd28 in combination with anti‐PD‐1 therapy, and targeting Treg and immunosuppressive myeloid cells may provide a mechanistic explanation at least partially for this effect.

In conclusion, the strategic, rational modification of synthetic lipid nanoparticles with 16:0 LPC creates a highly sophisticated, metabolism‐guided nanocarrier that specifically navigates the immunosuppressive TME. It possesses the unique capability to selectively deliver precise gene‐silencing therapeutics to tumor cell populations while actively evading the immune system. By utilizing this LPC‐LNPs, we successfully achieved cancer cell *Cd28* knockdown by *Cd28* siRNA in vivo, which extensively remodels TME to recruit and activate CD8^+^ T cell antitumor immune response, inhibit tumor growth, and overcome anti‐PD‐1 resistance. Our results offer valuable insights for developing cancer cell‐specific LNP as a useful tool for cancer immunotherapy. These findings possess profound clinical translatability, establishing a highly reliable, easily manufactured tool for precision oncological genetic manipulation, and definitively validating tumor‐intrinsic CD28 as a targetable molecule for breaking tumor immunoevasion and overcoming immunotherapy resistance.

## 4 Experimental Section

### 4.1 Materials

Heptadecan‐9‐yl 8‐((2‐hydroxyethyl)(6‐oxo‐6‐(undecyloxy)hexyl)amino)octanoate (SM‐102), 1,2‐Distearoyl‐sn‐glycero‐3‐phosphorylcholine (DSPC), DMG‐PEG2000, Cholesterol (CHO) and 1‐Palmitoyl‐sn‐glycero‐3‐phosphocholine (LPC 16:0) were purchased from Bidepharm (China). The DiR fluorescent dye and Quant‐iT RiboGreen RNA Reagent were obtained from Thermo Fisher Scientific (USA). Zombie‐Aqua, anti‐CD45‐APC‐Cy7, anti‐CD45‐BV785, anti‐CD19‐BV785, anti‐CD45‐BV650, anti‐CD3‐Percp‐Cy5.5, anti‐NK1.1‐PE‐Cy7, anti‐CD49b‐PE‐Cy7, anti‐CD4‐BV650, anti‐CD8a‐BV605, anti‐CD11b‐Percp‐Cy5.5, anti‐Ly6G‐BV421, anti‐Ly6G‐BV605, anti‐Ly6C‐PE, anti‐Ly6C‐BV650, anti‐F4/80‐APC, anti‐F4/80‐BV605, anti‐CD11c‐PE‐Cy7, anti‐MHC II‐APC‐Cy7, anti‐XCR1‐APC, anti‐XCR1‐BV421, anti‐EpCAM‐PE, anti‐CD31‐BV421, anti‐CD140a‐APC, anti‐CD28‐FITC, anti‐Granzyme B‐PE, anti‐Perforin‐FITC, anti‐IFNγ‐APC, anti‐TNFα‐BV421, anti‐IL2‐PE‐Cy5, anti‐FoxP3‐BV421, anti‐CD16/32 were purchased from BioLegend (USA). Anti‐CD28 (EPR22076) was purchased from Abcam (USA). Anti‐GAPDH was purchased from ProteinTech (China). InVivoMAb anti‐mouse PD‐1 (CD279) and InVivoMAb rat IgG2b isotype control were purchased from BioXCell (USA). The buffer and reagents for CyTOF were obtained from Standard BioTools (USA). Antibodies used for CyTOF (Tables S1 and S2) were purchased from Standard BioTools (USA), BioLegend (USA), and R&D Systems (USA).

### 4.2 Cell Culture and Animals

Mouse 4T1 triple‐negative breast cancer cells, mouse LLC Lewis lung cancer cells, and mouse B16F10 melanoma cells were obtained from ATCC (USA). 4T1 cells were cultured in RMPI 1640 with 100 IU mL^−1^ penicillin/streptomycin and 10% FBS (v/v) at 37°C. LLC and B16F10 cells were cultured in DMEM/F12 1:1 with 100 IU mL^−1^ penicillin/streptomycin and 10% FBS (v/v) at 37°C. Six‐to‐eight‐week‐old female BALB/c and C57BL/6J mice were obtained from Beijing Vital River Laboratory Animal Technology Co., Ltd (Beijing, China), and were housed in standard pathogen‐free conditions. All experiments were performed according to the Animal Welfare Act and other regulations associated with animals and experiments and were approved by Nankai University Laboratory Animal Welfare Ethics Review Committee (2022‐SYDWLL‐000405).

### 4.3 Synthesis of LNP

LNPs were prepared by vortex mixing or using a microfluidic device. For high‐throughput screening, lipids were dissolved in ethanol solution at a molar ratio of 50/10/38.5/1.5/x (x = 0, 6, 10, 14, 18, 22) for ionizable lipid (SM‐102), DSPC, cholesterol, DMG‐PEG2000, and three candidate lipids. RNA was dissolved in a citrate buffer (50.0 mM, pH 4.0). The ethanol and aqueous solutions were rapidly vortex‐mixed. For the preparation of COM‐LNPs and LPC‐LNPs, the molar ratio was 50/10/38.5/1.5/0 and 50/10/38.5/1.5/14 for SM‐102, DSPC, cholesterol, DMG‐PEG2000, and 16:0 LPC. The ethanol and aqueous solutions were mixed using a microfluidic device at a flow rate of 12 mL/minute, and an aqueous‐to‐ethanol volume ratio of 3:1. Dil‐labeled LNPs were prepared by adding Dil, accounting for 1.0 mol% of total lipids, into the ethanol phase before microfluidic mixing. The obtained LNPs were purified and concentrated using ultrafiltration centrifuge tubes (Amicon Ultra, MWCO 10 kDa). PBS containing 10% sucrose was used for buffer exchange to remove residual ethanol.

### 4.4 Characteristic of LNP

Characterization of particle size, polydispersity index (PDI), and zeta potential of the LNPs was performed using a Zetasizer Nano ZS (Malvern Panalytical). LNPs were diluted 100‐fold in ultrapure water and equilibrated at 25 °C for 120 s. For TEM imaging, 5 µL of the LNP sample was deposited onto a carbon‐coated grid and incubated for 10 min. Excess liquid was blotted with filter paper, followed by negative staining with 5 µL of 2% (w/v) uranyl acetate solution for 10 min. The grid was air‐dried at room temperature and observed using a Transmission Electron Microscope (Talos F200C, TESCAN). The encapsulation efficiency (EE%) was calculated using the Quant‐iT RiboGreen RNA assay (Invitrogen, R32700). Briefly, LNPs were diluted in 1× Tris‐EDTA (TE) buffer to measure the external RNA concentrations. LNPs were diluted in 1× TE buffer containing 1% (v/v) Triton X‐100 and incubated at room temperature for 15 min to disrupt the structure of LNPs. LNPs in TE buffer, LNPs in Triton X‐100, and standards were plated in black 96‐well plates. The RiboGreen reagent was added to each well and incubated for 15 min at room temperature in the dark. Fluorescence was measured using a microplate reader at an excitation wavelength of 480 nm and an emission wavelength of 520 nm. RNA concentrations were calculated from a standard curve prepared using the kit‐supplied RNA standards. EE% was calculated as EE (%)  =  [(Total RNA—External RNA)/Total RNA] × 100%.

### 4.5 Internalization Pathway of LNPs

4T1 cells were cultured in 12‐well plates with 1×10^5^ cells per well overnight. The cells were pretreated with 100 µM amiloride, 20 µM chlorpromazine, 200 µM genistein, and 80 µM dynasore for 1 h at 37 °C. Then, cells were treated with COM‐LNPs or LPC‐LNPs packaged with Cy5‐siRNA (1 µg/mL based on siRNA) for 4 h. The cellular uptake was measured by flow cytometry (Ex: 633 nm, Em: 640–670 nm).

### 4.6 Apparatus

Flow cytometry analysis was conducted using a BD LSRFortessa. Cancer cell sorting was performed using Sony MA900 Multi‐Application Cell Sorter. QPCR analysis was conducted using QuantStudio 7 Flex Real‐Time PCR System. FluidicLab Smart NP Generator S2 was used for LNP synthesis. CyTOF analysis was performed using Helios system.

### 4.7 LNP Delivery Efficacy Assay In Vitro

To assess the delivery efficacy of COM‐LNPs and LPC‐LNPs in cancer cells and immune cells in vitro, we synthesized COM‐LNPs and LPC‐LNPs modified with DiR fluorescent dye or packaged with GFP mRNA, Luciferase mRNA, or Cy5‐mRNA. 4T1 cells in complete medium were treated with indicated LNP for indicated time followed by flow cytometry analysis. For measuring delivery efficacy in splenocytes, spleens from C57BL/6J mice were macerated through a 40 µm Nylon Cell Strainer (Corning Life Sciences) followed by red blood cell removal via red blood cell lysis buffer (Solarbio) to generate a single‐cell splenocytes suspension. Splenocytes were seeded on 6‐well plates in complete medium and treated with Dil‐LNP containing 1 µg GFP mRNA per well for 24 h. Two panels of antibodies were used for profiling LNP delivery efficiency in different cell types. One panel containing Zombin‐BV510, anti‐CD45‐APC‐Cy7, anti‐CD3‐Percp‐Cy5.5, anti‐CD4‐BV650, anti‐CD8a‐BV605, anti‐NK1.1‐PE‐Cy7, anti‐CD19‐BV785 for lymphoid cells. Another panel containing Zombin‐BV510, anti‐CD45‐BV785, anti‐CD11b‐Percp‐Cy5.5, anti‐Ly6C‐BV650, anti‐Ly6G‐BV421, anti‐F4/80‐BV605, anti‐CD11c‐PE‐Cy7, anti‐MHC II‐APC‐Cy7, anti‐XCR1‐APC was used for profiling myeloid cells. Briefly, cells were incubated for 10 min with FcR blocking anti‐CD16/32 antibody, and then incubated with the indicated antibodies for 20 min protected from light. Freshly generated splenocytes without LNP treatment were used as negative control. Cells were then analyzed by BD LSRFortessa. Flow cytometry data were analyzed using the FlowJo_V10 software.

### 4.8 LNP Delivery Efficacy Assay In Vivo

To assess the delivery efficacy of COM‐LNPs and LPC‐LNPs in cancer cells and immune cells in vivo, we synthesized COM‐LNPs and LPC‐LNPs packaged with GFP mRNA. LNPs containing 20 µg mRNA in 50 µL volume were intratumorally injected into 4T1 tumors at day 10 after tumor inoculation, when tumor volume reached 50 mm^3^. Tumor‐bearing mice were sacrificed 24 h after injection. Tumor tissues were dissociated into single‐cell suspension, and GFP expression profile was assayed by flow cytometry. Briefly, two panels of antibodies were adopted. One panel containing Zombin‐BV510, anti‐CD45‐APC‐Cy7, anti‐CD3‐Percp‐Cy5.5, anti‐CD4‐BV650, anti‐CD8a‐BV605, anti‐CD49b‐PE‐Cy7, anti‐CD19‐BV785, anti‐EpCAM‐PE, anti‐CD31‐BV421 and anti‐CD140a‐APC for lymphoid cells, cancer cells, endothelial cells, and fibroblasts. Another panel containing Zombin‐BV510, anti‐CD45‐BV650, anti‐CD11b‐Percp‐Cy5.5, anti‐Ly6C‐PE, anti‐Ly6G‐BV421, anti‐F4/80‐BV605, anti‐CD11c‐PE‐Cy7, anti‐MHC II‐APC‐Cy7, anti‐XCR1‐APC was used for profiling myeloid cells. Dissociated single cells were incubated for 10 min with FcR‐blocking anti‐CD16/32 antibody, and then incubated with the indicated antibodies for 20 min protected from light. Cells were then analyzed by BD LSRFortessa. Flow cytometry data were analyzed using the FlowJo_V10 software.

### 4.9 RNA Interfering

Small interfering RNA (siRNA) of mouse *Lpcat1*, *Lpcat2*, *Lpcat3*, and *Lpcat4* were transfected with LIPOFECTAMINE RNAIMAX (13778150, Invitrogen) according to the standard protocol. The efficiency of indicated gene knockdown was verified by Western blot.

### 4.10 RNA Extraction and Quantitative Real‐Time PCR (Q‐PCR)

RNA extraction from cells was performed using RNAfast2000 kit (Fastagen) according to the manufacturer's instructions and quantified using NanoDrop 2000. Then, reverse transcription of RNA into DNA was completed using the RT Master Mix (TOYOBO) at 20 µL volume for 2 µg RNA. Quantitative real‐time PCR using Rapid Taq Master Mix (Vazyme Biotech) was performed on QuantStudio 7 Flex Real‐Time PCR System (Applied Biosystems) for 40 cycles. Mouse *Actb* was used as a housekeeping gene. Relative expression of targeted genes was calculated by 2^−ΔΔCt^. Following primers were used for QPCR analysis: *Actb* forward: 5‘‐GGCTGTATTCCCCTCCATCG‐3‘; *Actb* reverse: 5‘‐CCAGTTGGTAACAATGCCATGT‐3‘; *Cd28* forward: 5‘‐GTTCTTGGCTCTCAACTTCTTCT‐3‘; *Cd28* reverse: 5‘‐TGAGGCTGACCTCGTTGCTAT‐3‘; *Cd274* forward: 5‘‐GCTCCAAAGGACTTGTACGTG‐3‘; *Cd274* reverse: 5‘‐TGATCTGAAGGGCAGCATTTC‐3‘; *Lpcat1* forward: 5‘‐AGAGCAGAGACATCCCAATCT‐3‘; *Lpcat1* reverse: 5‘‐CCAGGTTTGAAGGTAATGAGGC‐3‘; *Lpcat2* forward: 5‘‐CCCTTCGTCCAGCAGACTAC‐3‘; *Lpcat2* reverse: 5‘‐GCAGCAAAATTATTCCAACCAGT‐3‘; *Lpcat3* forward: 5‘‐GACGGGGACATGGGAGAGA‐3‘; *Lpcat3* reverse: 5‘‐GTAAAACAGAGCCAACGGGTAG‐3‘; *Lpcat4* forward: 5‘‐GACCCCACCCTTTATGCCAA‐3‘; *Lpcat4* reverse: 5‘‐TGGCTGATCATTCGACTCCG‐3‘.

### 4.11 Western Blot

Western blot was performed following the routine procedures. Briefly, whole‐cell protein lysate was prepared using cell lysis buffer (Cell Signaling Technology) supplemented with Complete Protease Inhibitor Cocktail (Roche) after incubation on ice for 15 min and centrifugation at 14 400 rpm for 20 min at 4 °C. Protein concentration measuring was conducted using the Pierce BCA Protein Assay Kit (Thermo Fisher Scientific). 25 ng of total protein from each sample was loaded for Western blot analysis using 12% gel and transferred under 220 mA for 2 h. 5% BSA was used for blockade for 1 h at room temperature. Primary antibodies and their dilutions were indicated as follows: CD28 (1:1000; Abcam), LPCAT1 (1:1000; Proteintech), LPCAT2 (1:1000, Abclonal), LPCAT3 (1:1000, Abclonal), LPCAT4 (1:1000, Abclonal) and GAPDH (1:2000; WanleiBio). Primary antibodies were incubated overnight at 4 °C. After washing by TBST for 10 min for 3 times, anti‐rabbit horseradish peroxidase (HRP) secondary antibody (Absin) was used at 1:2000 dilution and incubated for 1 h at room temperature. After washing with TBST for 10 min for 3 times, membranes were visualized by SuperSignal West Femto Maximum Sensitivity Substrate (Thermo Scientific) and detected using Amersham Imager 600 (General Electric).

### 4.12 Tumor Model

For tumor inoculation, 6–8 week female BALB/c mice were orthotopically inoculated with 1×10^6^ 4T1 cells into the fourth mammary gland pad for the establishment of mouse TNBC tumor model, and 6–8 week female C57BL/6J mice were subcutaneously inoculated with 1×10^6^ LLC cells or 5×10^5^ B16F10 cells for establishment of mouse lung cancer or melanoma tumor model. Tumor volume was monitored every 3 days. Tumor size was calculated by following formula: V = Length×Width^2^/2. Tumor‐bearing mice were sacrificed when tumor volume reached 2000 mm^3^.

For LNP treatment, LNPs packaged with 20 µg *Cd28* siRNA in 50 µL volume were intratumorally injected at day 10, 12, and 14 after tumor inoculation. Control group injected LNP capsuled with normal control sequence. For LNP and anti‐PD‐1 combined treatment, LPC‐LNPs with Cd28 siRNA or normal control sequence was administrated as mentioned above. Anti‐PD‐1/Isotype control IgG2a were intraperitoneally injected at day 9, 12, 15 and 18 after tumor inoculation, with 200 µg antibody in 100 µL PBS each time.

### 4.13 Tumor Tissue Dissociation for Single‐Cell Suspension

Tumor‐bearing mice were euthanized and dissected at the end of the experiment to collect tumor tissues. Tumor tissues were cut into pieces about 1 mm and enzymatically digested with 1 mg/mL collagenase I (Gibco), 1 mg/mL collagenase IV (Gibco), and 300 µg/mL DNase I (Sigma–Aldrich) at 37 °C for 40 min, with the suspension mixed every 10 min. The suspension was then filtered through a 40 µm Nylon Cell Strainer (Corning Life Sciences) and centrifuged at 500 g for 5 min. Erythrocytes were removed by hypotonic lysis for 5 min at 4 °C. Cell pellets were then resuspended in PBS and the cell density was adjusted as required for cancer cell sorting, flow cytometry, or CyTOF analysis.

### 4.14 Cancer Cell Sorting

Single‐cell suspension was incubated for 10 min with FcR‐blocking anti‐CD16/32 antibody, and then incubated with indicated antibodies for 20 min protected from light. CD45^−^EpCAM^+^ cancer cells were then analyzed and sorted using Sony MA900 Multi‐Application Cell Sorter. Sorted cells were used for RNA extraction.

### 4.15 Flow Cytometry Analysis for Tumor Microenvironment

Three panels of antibodies were adopted for deeply profiling lymphoid cells, Treg, and myeloid cells. Panel 1 for lymphoid cells: Zombin‐BV510, anti‐CD45‐APC‐Cy7, anti‐CD3‐Percp‐Cy5.5, anti‐CD4‐BV650, anti‐CD8a‐BV605, anti‐CD49b‐PE‐Cy7, anti‐CD19‐BV785, anti‐IFNγ‐APC, anti‐TNFα‐BV421, anti‐Granzyme B‐PE and anti‐Perforin‐FITC. Panel 2 for Treg: Zombin‐BV510, anti‐CD45‐APC‐Cy7, anti‐CD3‐Percp‐Cy5.5, anti‐CD4‐BV650, anti‐CD8a‐BV605, anti‐CD49b‐PE‐Cy7 and anti‐FoxP3‐PE. Panel 3 for myeloid cells: Zombie‐BV510, anti‐CD45‐BV650, anti‐CD11b‐Percp‐Cy5.5, anti‐Ly6C‐PE, anti‐Ly6G‐BV605, anti‐F4/80‐APC, anti‐CD11c‐PE‐Cy7, anti‐MHC II‐APC‐Cy7 and anti‐XCR1‐BV421. Dissociated single cells were incubated for 10 min with FcR‐blocking mouse anti‐CD16/32 antibody, followed with incubation with indicated antibodies for 20 min protected from light. For intracellular staining, cells were fixed in 4% polymerized formaldehyde for 20 min, and then penetrated in permeabilization buffer (BioLegend) and stained with indicated intracellular antibodies. For nuclear staining, cells were fixed and permeabilized with True‐Nuclear Transcription Factor Buffer Set (BioLegend). Briefly, cells were fixed with Fixation buffer for 40 min at 4 °C on a rotator, followed by twice washing with permeabilization buffer and stained with indicated nuclear antibodies overnight at 4 °C on a rotator. Cells were then analyzed by BD LSRFortessa. Flow cytometry data were analyzed using the FlowJo_V10 software.

### 4.16 CyTOF Analysis for Tumor Microenvironment

CyTOF analysis was performed as previously described [77]. In brief, 3×10^6^ cells per sample were incubated with Cell‐ID Cisplatin (Standard BioTools) at a final concentration of 0.5 mM in 1 mL PBS for 2 min to distinguish live and dead cells, and were stopped by washing with MaxPar Cell Staining Buffer (Standard BioTools). Cells were then washed, fixed using 1.6% polyformaldehyde, and permeabilized with freshly prepared 1× Barcode Perm Buffer in PBS (Standard BioTools). Cells were further incubated with Palladium barcodes (Standard BioTools) for 30 min at room temperature. Cells were subsequently washed twice with MaxPar Cell Staining Buffer (Standard BioTools) and pooled together. Pooled cells were resuspended with PBS containing Fc receptor blocking mouse anti‐CD16/32 antibody (BioLegend) for 10 min and stained with a cocktail of surface staining antibodies for 30 min at room temperature. After washing with MaxPar Cell Staining Buffer twice, cells were fixed for 15 min in Fix I buffer (Standard BioTools), followed by washing twice with freshly prepared Perm‐S Buffer (Standard BioTools). Cells were stained with a cocktail of intracellular staining antibodies for 30 min at room temperature and then washed twice with MaxPar Cell Staining Buffer. Cells were fixed and permeabilized using True‐Nuclear Transcription Factor Buffer Set (BioLegend) as instructed. Then cells were stained with nuclear‐staining antibodies for 30 min at room temperature and washed twice with MaxPar Cell Staining Buffer, followed by fixing with MaxPar Fix and Perm Buffer containing 0.125 mM Cell‐ID Intercalator‐Ir (Standard BioTools) at room temperature for 15 min to stain the nuclei. For acquisition, cells were washed twice with MaxPar Cell Staining Buffer and deionized water and then resuspended to 1×10^6^ cells/mL in Cell Acquisition Solution (Standard BioTools) with a 1:10 dilution of EQ Four Element Beads (Standard BioTools). The cells were introduced to Helios system (Standard BioTools) at an event rate of < 500 events/second. Upstream analysis of manually gating for immune cells was performed using FlowJo_V10 software. The downstream analysis including clustering via PhenoGraph algorithm and dimension reduction by UMAP was accomplished using R. All antibodies used in CyTOF analysis are summarized in Tables S1 and S2.

### 4.17 Bioinformatics Analysis of Lands’ Cycle Geneset in Human Public scRNAseq Datasets

Human breast cancers (GEO: GSE176078), TNBC (European Genome‐phenome Archive: EGAS00001004809), and NSCLC (GEO: GSE207422) were downloaded for subsequent analysis. Cell identity was identified based on available metadata or manually annotation by unsupervised clustering with Seurat package. Lands‘ cycle geneset included *LPCAT1, LPCAT2, LPCAT3, LPCAT4, ACSL3, FASN* and *SDC1*. AddModuleScore function in Seurat package was utilized for scoring of Lands’ cycle geneset. DotPlot function in Seurat package was utilized for visualization of *PLA2G4A, PLA2G4B, PLA2G4C* and Lands‘ cycle geneset scoring.

### 4.18 Statistical Analysis

Statistical analysis was performed using unpaired t‐test, one‐way analysis of variance (ANOVA) with Tukey's multiple comparison for comparisons between multiple groups, and two‐way ANOVA for tumor growth curves. Survival curves were constructed using Kaplan–Meier estimates and tested using the log‐rank test. GraphPad Prism software (version 8.0) was used for all analyses. The significance levels were set at *p < 0.05 for statistical significance, **p < 0.01, ***p < 0.001, ****p < 0.0001. *p* values > 0.05 were considered not significant (ns). All data are presented as means ± standard deviation (SD) or standard error of the mean (Sem) (n ≥ 3).

## Author Contributions

X.C. and D.D. designed the experimental approach and supervised the study; K.W. and S.J. screened, synthesized, and characterized the LNP. Y.C., K.W., and J.F. verified the specificity of LNP in vitro and in vivo. Y.C., J.F., Y.S., W.G., X.L., J.L., Z.C., Y.Q., and X.S.C. performed the immunological analyses and the animal experiments. Y.C., K.W., D.D., and X.C. analyzed data and wrote the paper with input from all authors.

## Funding

This work was supported by grants from the National Natural Science Foundation of China (82388201, 82302077) and the Chinese Academy of Medical Sciences Innovation Fund for Medical Sciences (2025‐I2M‐XHZY‐019 and 2024‐I2M‐ZD‐005).

## Ethics Statement

No written consent has been obtained from the patients, as there is no patient‐identifiable data included in this study. No clinical trials are involved in this study. All animal experiments were performed according to the Animal Welfare Act and other regulations associated with animals and experiments and were approved by Nankai University Laboratory Animal Welfare Ethics Review Committee (2022‐SYDWLL‐000405).

## Conflicts of Interest

The authors declare no conflicts of interest.

## Supporting information




**Supporting File**: advs76003‐sup‐0001‐SuppMat.docx.

## Data Availability

The data that support the findings of this study are available on request from the corresponding author. The data are not publicly available due to privacy or ethical restrictions.

## References

[1] A. J. Korman , S. C. Garrett‐Thomson , and N. Lonberg , “The Foundations of Immune Checkpoint Blockade and the ipilimumab Approval Decennial,” Nature Reviews Drug Discovery 21, no. 7 (2021): 509–528, 10.1038/s41573-021-00345-8. 34937915

[2] Z.‐X. Wang , F. Chen , M.‐M. He , et al., “Optimizing Immunotherapy for Gastrointestinal Tract Cancers: Clinical Progress and Perspectives,” Immunity & Inflammation 2, no. 1 (2026): 10, 10.1007/s44466-026-00025-5.

[3] A. J. Schoenfeld and M. D. Hellmann , “Acquired Resistance to Immune Checkpoint Inhibitors,” Cancer Cell 37, no. 4 (2020): 443–455, 10.1016/j.ccell.2020.03.017. 32289269 PMC7182070

[4] M. D. Vesely , T. Zhang , and L. Chen , “Resistance Mechanisms to Anti‐PD Cancer Immunotherapy,” Annual Review of Immunology 40 (2022): 45–74, 10.1146/annurev-immunol-070621-030155. 35471840

[5] Y. Zong , Y. Lin , T. Wei , Q. Cheng , and L. Nanoparticle , “Lipid Nanoparticle (LNP) Enables mRNA Delivery for Cancer Therapy,” Advanced Materials 35, no. 51 (2023): 2303261, 10.1002/adma.202303261. 37196221

[6] X. Hou , T. Zaks , R. Langer , and Y. Dong , “Lipid Nanoparticles for mRNA Delivery,” Nature Reviews Materials 6, no. 12 (2021): 1078–1094, 10.1038/s41578-021-00358-0. 34394960 PMC8353930

[7] P. R. Cullis and P. L. Felgner , “The 60‐year evolution of lipid nanoparticles for nucleic acid delivery,” Nature Reviews Drug Discovery 23, no. 9 (2024): 709–722, 10.1038/s41573-024-00977-6. 38965378

[8] R. H. Fang , W. Gao , and L. Zhang , “Targeting Drugs to Tumours Using Cell Membrane‐coated Nanoparticles,” Nature Reviews Clinical Oncology 20, no. 1 (2023): 33–48, 10.1038/s41571-022-00699-x. 36307534

[9] P. Tarantino , B. Ricciuti , S. M. Pradhan , and S. M. Tolaney , “Optimizing the safety of antibody–drug conjugates for patients With solid tumours,” Nature Reviews Clinical Oncology 20, no. 8 (2023): 558–576, 10.1038/s41571-023-00783-w. 37296177

[10] M. T. Lotze , S. H. Olejniczak , and D. Skokos , “CD28 co‐stimulation: Novel Insights and Applications in Cancer Immunotherapy,” Nature Reviews Immunology 24, no. 12 (2024): 878–895, 10.1038/s41577-024-01061-1. PMC11598642 39054343

[11] A. Bates , N. E. Fletcher , F. Gao , M. Pereira Pinho , and T. Dong , “Leveraging T Cells for Cancer Immunotherapy,” Immunity & Inflammation 1, no. 1 (2025): 9, 10.1007/s44466-025-00007-z. 41669116 PMC12883538

[12] E. Hui , J. Cheung , J. Zhu , et al., “T cell costimulatory receptor CD28 is a primary target for PD‐1–mediated inhibition,” Science 355, no. 6332 (2017): 1428–1433, 10.1126/science.aaf1292. 28280247 PMC6286077

[13] A. O. Kamphorst , A. Wieland , T. Nasti , et al., “Rescue of exhausted CD8 T cells by PD‐1–targeted therapies is CD28‐dependent,” Science 355, no. 6332 (2017): 1423–1427, 10.1126/science.aaf0683. 28280249 PMC5595217

[14] K. M. Cappell and J. N. Kochenderfer , “A Comparison of Chimeric Antigen Receptors Containing CD28 versus 4‐1BB Costimulatory Domains,” Nature Reviews Clinical Oncology 18, no. 11 (2021): 715–727, 10.1038/s41571-021-00530-z. 34230645

[15] Z. Yang , X. Liu , J. Zhu , et al., “Inhibiting Intracellular CD28 in Cancer Cells Enhances Antitumor Immunity and Overcomes Anti‐PD‐1 Resistance via Targeting PD‐L1,” Cancer Cell 43, no. 1 (2025): 86–102.e10, 10.1016/j.ccell.2024.11.008. 39672166

[16] Q. Tang and A. Khvorova , “RNAi‐based Drug Design: Considerations and Future Directions,” Nature Reviews Drug Discovery 23, no. 5 (2024): 341–364, 10.1038/s41573-024-00912-9. 38570694 PMC11144061

[17] M. Moazzam , M. Zhang , A. Hussain , X. Yu , J. Huang , and Y. Huang , “The Landscape of Nanoparticle‐based siRNA Delivery and Therapeutic Development,” Molecular Therapy 32, no. 2 (2024): 284–312, 10.1016/j.ymthe.2024.01.005. 38204162 PMC10861989

[18] A. Hoffmann , G. Cheng , and D. Baltimore , “NF‐κB: Master Regulator of Cellular Responses in Health and Disease,” Immunity & Inflammation 1, no. 1 (2025): 2, 10.1007/s44466-025-00014-0. 41669117 PMC12883533

[19] T. L. Hunter , Y. Bao , Y. Zhang , et al., “In vivo CAR T Cell Generation to Treat Cancer and Autoimmune Disease,” Science 388, no. 6753 (2025): 1311–1317, 10.1126/science.ads8473. 40536974

[20] J. A. Katakowski , G. Mukherjee , S. E. Wilner , et al., “Delivery of siRNAs to Dendritic Cells Using DEC205‐Targeted Lipid Nanoparticles to Inhibit Immune Responses,” Molecular Therapy 24, no. 1 (2015): 146–155, 10.1038/mt.2015.175. 26412590 PMC4754549

[21] J. F. Bimbo , E. van Diest , D. E. Murphy , et al., “T Cell‐specific Non‐viral DNA Delivery and in vivo CAR‐T Generation Using Targeted Lipid Nanoparticles,” Journal for ImmunoTherapy of Cancer 13, no. 7 (2025): 011759, 10.1136/jitc-2025-011759. PMC12258353 40659448

[22] Q. Cheng , T. Wei , L. Farbiak , L. T. Johnson , S. A. Dilliard , and D. J. Siegwart , “Selective organ targeting (SORT) nanoparticles for tissue‐specific mRNA delivery and CRISPR–Cas gene editing,” Nature Nanotechnology 15, no. 4 (2020): 313–320, 10.1038/s41565-020-0669-6. PMC7735425 32251383

[23] M. Martin‐Perez , U. Urdiroz‐Urricelqui , C. Bigas , and S. A. Benitah , “The Role of Lipids in Cancer Progression and Metastasis,” Cell Metabolism 34, no. 11 (2022): 1675–1699, 10.1016/j.cmet.2022.09.023. 36261043

[24] B. Wang and P. Tontonoz , “Phospholipid Remodeling in Physiology and Disease,” Annual Review of Physiology 81 (2019): 165–188, 10.1146/annurev-physiol-020518-114444. PMC7008953 30379616

[25] K. Wang , C.‐W. Lee , X. Sui , et al., “The Structure of Phosphatidylinositol Remodeling MBOAT7 Reveals Its Catalytic Mechanism and Enables Inhibitor Identification,” Nature Communications 14, no. 1 (2023): 3533, 10.1038/s41467-023-38932-5. PMC10267149 37316513

[26] S. Wilhelm , A. J. Tavares , Q. Dai , et al., “Analysis of Nanoparticle Delivery to Tumours,” Nature Reviews Materials 1, no. 5 (2016): 16014, 10.1038/natrevmats.2016.14.

[27] D. Rosenblum , N. Joshi , W. Tao , J. M. Karp , and D. Peer , “Progress and Challenges towards Targeted Delivery of Cancer Therapeutics,” Nature Communications 9, no. 1 (2018): 1410, 10.1038/s41467-018-03705-y. PMC5897557 29650952

[28] J. Liu , Y. Chen , and L. O'Neill , “Phospholipid Metabolism in Innate Immunity and Inflammation: From Basic to Clinic,” Immunity & Inflammation 1, no. 1 (2025): 6, 10.1007/s44466-025-00001-5.

[29] A. Bassez , H. Vos , L. Van Dyck , et al., “A Single‐cell Map of Intratumoral Changes During Anti‐PD1 Treatment of Patients With Breast Cancer,” Nature Medicine 27, no. 5 (2021): 820–832, 10.1038/s41591-021-01323-8. 33958794

[30] S. Z. Wu , G. Al‐Eryani , D. L. Roden , et al., “A Single‐cell and Spatially Resolved Atlas of human Breast Cancers,” Nature Genetics 53, no. 9 (2021): 1334–1347, 10.1038/s41588-021-00911-1. 34493872 PMC9044823

[31] J. Hu , L. Zhang , H. Xia , et al., “Tumor Microenvironment Remodeling After Neoadjuvant Immunotherapy in Non‐small Cell Lung Cancer Revealed by Single‐cell RNA Sequencing,” Genome Medicine 15, no. 1 (2023): 15, 10.1186/s13073-023-01164-9. 36869384 PMC9985263

[32] C. Bartolacci , C. Andreani , G. Vale , et al., “Targeting De Novo Lipogenesis and the Lands Cycle Induces Ferroptosis in KRAS‐mutant Lung Cancer,” Nature Communications 13, no. 1 (2022): 4327, 10.1038/s41467-022-31963-4. PMC9325712 35882862

[33] M. Koivusalo , C. Welch , H. Hayashi , et al., “Amiloride Inhibits Macropinocytosis by Lowering Submembranous pH and Preventing Rac1 and Cdc_42_ Signaling,” Journal of Cell Biology 188, no. 4 (2010): 547–563, 10.1083/jcb.200908086. 20156964 PMC2828922

[34] J. Rejman , A. Bragonzi , and M. Conese , “Role of Clathrin‐ and Caveolae‐mediated Endocytosis in Gene Transfer Mediated by Lipo‐ and Polyplexes,” Molecular Therapy 12, no. 3 (2005): 468–474, 10.1016/j.ymthe.2005.03.038. 15963763

[35] G. A. Kumar , J. Karmakar , C. Mandal , and A. Chattopadhyay , “Leishmania donovani Internalizes Into Host Cells via Caveolin‐mediated Endocytosis,” Scientific Reports 9, no. 1 (2019): 12636, 10.1038/s41598-019-49007-1. 31477757 PMC6718660

[36] M. Furmanik , M. Chatrou , R. van Gorp , et al., “Reactive Oxygen‐Forming Nox5 Links Vascular Smooth Muscle Cell Phenotypic Switching and Extracellular Vesicle‐Mediated Vascular Calcification,” Circulation Research 127, no. 7 (2020): 911–927, 10.1161/CIRCRESAHA.119.316159. 32564697

[37] E. Blanco , H. Shen , and M. Ferrari , “Principles of Nanoparticle Design for Overcoming Biological Barriers to Drug Delivery,” Nature Biotechnology 33, no. 9 (2015): 941–951, 10.1038/nbt.3330. PMC4978509 26348965

[38] I. V. Zelepukin , K. G. Shevchenko , and S. M. Deyev , “Rediscovery of Mononuclear Phagocyte System Blockade for Nanoparticle Drug Delivery,” Nature Communications 15, no. 1 (2024): 4366, 10.1038/s41467-024-48838-5. PMC11111695 38777821

[39] Z. Cao , X. Liu , W. Zhang , et al., “Biomimetic Macrophage Membrane‐Camouflaged Nanoparticles Induce Ferroptosis by Promoting Mitochondrial Damage in Glioblastoma,” ACS Nano 17, no. 23 (2023): 23746–23760, 10.1021/acsnano.3c07555. 37991252 PMC10722604

[40] T. D. Wu , S. Madireddi , P. E. de Almeida , et al., “Peripheral T Cell Expansion Predicts Tumour Infiltration and Clinical Response,” Nature 579, no. 7798 (2020): 274–278, 10.1038/s41586-020-2056-8. 32103181

[41] X. Wang , C. Tokheim , S. S. Gu , et al., “In vivo CRISPR screens identify the E3 ligase Cop1 as a modulator of macrophage infiltration and cancer immunotherapy target,” Cell 184, no. 21 (2021): 5357–5374.e22, 10.1016/j.cell.2021.09.006. 34582788 PMC9136996

[42] I. Sagiv‐Barfi , H. E. K. Kohrt , D. K. Czerwinski , P. P. Ng , B. Y. Chang , and R. Levy , “Therapeutic Antitumor Immunity by Checkpoint Blockade Is Enhanced by Ibrutinib, an Inhibitor of both BTK and ITK,” Proceedings of the National Academy of Sciences 112, no. 9 (2015): 966–972, 10.1073/pnas.1500712112. PMC4352777 25730880

[43] H. Lin , I. Kryczek , S. Li , et al., “Stanniocalcin 1 Is a Phagocytosis Checkpoint Driving Tumor Immune Resistance,” Cancer Cell 39, no. 4 (2021): 480–493, 10.1016/j.ccell.2020.12.023. 33513345 PMC8044011

[44] D. Chen , Z. Jin , H. Chu , et al., “DNASE1L3‐expressing dendritic cells promote CD8+ T cell function and anti‐PD‐(L)1 therapy efficacy by degrading neutrophil extracellular traps,” Cancer Cell 43, no. 9 (2025): 1758–1775, 10.1016/j.ccell.2025.07.014. 40816293

[45] L. Gong , J. Luo , E. Yang , et al., “Cancer Immunology Data Engine Reveals Secreted AOAH as a Potential Immunotherapy,” Cell 188, no. 18 (2025): 5062–5080.e32, 10.1016/j.cell.2025.07.004. 40730154 PMC12434699

[46] J. H. Shin , H. B. Park , Y. M. Oh , et al., “Positive Conversion of Negative Signaling of CTLA4 Potentiates Antitumor Efficacy of Adoptive T‐cell Therapy in Murine Tumor Models,” Blood 119, no. 24 (2012): 5678–5687, 10.1182/blood-2011-09-380519. 22538857

[47] B. Xie , B. Yuan , X. Zhao , et al., “Ms4a7 expression in cDC1s Determines Cross‐presentation and Antitumor Immunity,” Science 390, no. 6774 (2025): ady5362, 10.1126/science.ady5362. 41231994

[48] X. Wu , B. Pan , C. Chu , et al., “CXCL16/CXCR6/TGF‐β Feedback Loop Between M‐MDSCs and Treg Inhibits Anti‐Bacterial Immunity During Biofilm Infection,” Advanced Science 12, no. 7 (2025): 2409537, 10.1002/advs.202409537. 39716908 PMC11831521

[49] J. H. Levine , E. F. Simonds , S. C. Bendall , et al., “Data‐Driven Phenotypic Dissection of AML Reveals Progenitor‐Like Cells That Correlate With Prognosis,” Cell 162, no. 1 (2015): 184–197, 10.1016/j.cell.2015.05.047. 26095251 PMC4508757

[50] E. Becht , L. McInnes , J. Healy , et al., “Dimensionality Reduction for Visualizing Single‐cell Data Using UMAP,” Nature Biotechnology 37, no. 1 (2019): 38–44, 10.1038/nbt.4314. 30531897

[51] G. Cheng , X. Yuan , M. S. Tsai , E. R. Podack , A. Yu , and T. R. Malek , “IL‐2 Receptor Signaling Is Essential for the Development of Klrg1+ Terminally Differentiated T Regulatory Cells,” The Journal of Immunology 189, no. 4 (2012): 1780–1791, 10.4049/jimmunol.1103768. 22786769 PMC3411868

[52] C. Y. C. Lee , B. C. Kennedy , N. Richoz , et al., “Tumour‐retained Activated CCR7+ Dendritic Cells Are Heterogeneous and Regulate Local Anti‐tumour Cytolytic Activity,” Nature Communications 15, no. 1 (2024): 682, 10.1038/s41467-024-44787-1. PMC10808534 38267413

[53] A. Kalbasi and A. Ribas , “Tumour‐intrinsic Resistance to Immune Checkpoint Blockade,” Nature Reviews Immunology 20, no. 1 (2020): 25–39, 10.1038/s41577-019-0218-4. PMC8499690 31570880

[54] J. Lei , K. Yang , W. Cao , et al., “Pancreatic‐targeted Lipid Nanoparticles Based on Organ Capsule Filtration,” Nature 652, no. 8108 (2026): 220–229, 10.1038/s41586-026-10158-7. 41741655

[55] R. Das , E. A. Halabi , I. R. Fredrich , et al., “Hybrid LNP Prime Dendritic Cells for Nucleotide Delivery,” Advanced Science 10, no. 33 (2023): 2303576, 10.1002/advs.202303576. 37814359 PMC10667837

[56] G. Zhao , L. Xue , H. C. Geisler , et al., “Precision Treatment of Viral Pneumonia Through Macrophage‐targeted Lipid Nanoparticle Delivery,” Proceedings of the National Academy of Sciences 121, no. 7 (2024): 2314747121, 10.1073/pnas.2314747121. PMC10873611 38315853

[57] S. H. Kiaie , H. Salehi‐Shadkami , S. M. S. Sajadi , B. Gharehchelou , and A. R. Zangi , “Antibody Targeted Delivery of Lipid Nanoparticles for RNA Therapeutics to Immune Cells,” International Journal of Biological Macromolecules 320 (2025): 145854, 10.1016/j.ijbiomac.2025.145854. 40675287

[58] J. Gilleron , W. Querbes , A. Zeigerer , et al., “Image‐based analysis of lipid nanoparticle–mediated siRNA delivery, intracellular trafficking and endosomal escape,” Nature Biotechnology 31, no. 7 (2013): 638–646, 10.1038/nbt.2612. 23792630

[59] G. Sahay , W. Querbes , C. Alabi , et al., “Efficiency of siRNA Delivery by Lipid Nanoparticles Is Limited by Endocytic Recycling,” Nature Biotechnology 31, no. 7 (2013): 653–658, 10.1038/nbt.2614. PMC3814166 23792629

[60] Y. Wang , Y. Chai , Y. Liu , et al., “Inhibition of Tumor Cell Macropinocytosis Driver DHODH Reverses Immunosuppression and Overcomes Anti‐PD1 Resistance,” Immunity 58, no. 10 (2025): 2456–2471.e6, 10.1016/j.immuni.2025.07.013. 40816268

[61] P. Guo , D. Liu , K. Subramanyam , et al., “Nanoparticle Elasticity Directs Tumor Uptake,” Nature Communications 9, no. 1 (2018): 130, 10.1038/s41467-017-02588-9. PMC5760638 29317633

[62] N. Kurabe , T. Hayasaka , M. Ogawa , et al., “Accumulated phosphatidylcholine (16:0/16:1) in human colorectal cancer; possible involvement of LPCAT_4_ ,” Cancer Science 104, no. 10 (2013): 1295–1302, 10.1111/cas.12221. 23815430 PMC7656554

[63] M. Tao , J. Luo , T. Gu , et al., “LPCAT1 reprogramming Cholesterol Metabolism Promotes the Progression of Esophageal Squamous Cell Carcinoma,” Cell Death & Disease 12, no. 9 (2021): 845, 10.1038/s41419-021-04132-6. 34518524 PMC8438019

[64] Y. Du , Q. Wang , X. Zhang , et al., “Lysophosphatidylcholine Acyltransferase 1 Upregulation and Concomitant Phospholipid Alterations in Clear Cell Renal Cell Carcinoma,” Journal of Experimental & Clinical Cancer Research 36, no. 1 (2017): 66, 10.1186/s13046-017-0525-1. 28494778 PMC5427523

[65] S. Chatterjee , E. Kon , P. Sharma , and D. Peer , “Endosomal Escape: A Bottleneck for LNP‐mediated Therapeutics,” Proceedings of the National Academy of Sciences 121, no. 11 (2024): 2307800120, 10.1073/pnas.2307800120. PMC10945858 38437552

[66] F. Michel , G. Attal‐Bonnefoy , G. Mangino , S. Mise‐Omata , and O. Acuto , “CD28 as a Molecular Amplifier Extending TCR Ligation and Signaling Capabilities,” Immunity 15, no. 6 (2001): 935–945, 10.1016/S1074-7613(01)00244-8. 11754815

[67] X. Pan , Y. Pan , Y. Su , et al., “Chronic TCR Signaling‐driven Suppression of the FOXO1‐KLHL6 Axis Promotes T Cell Exhaustion,” Immunity & Inflammation 2, no. 1 (2026): 8, 10.1007/s44466-025-00023-z. 41541249 PMC12799747

[68] C. J. Wang , F. Heuts , V. Ovcinnikovs , et al., “CTLA‐4 Controls Follicular Helper T‐cell Differentiation by Regulating the Strength of CD28 Engagement,” Proceedings of the National Academy of Sciences 112, no. 2 (2014): 524–529, 10.1073/pnas.1414576112. PMC4299196 25548162

[69] C. Cui , J. Wang , E. Fagerberg , et al., “Neoantigen‐driven B cell and CD4 T follicular helper cell collaboration promotes anti‐tumor CD8 T cell responses,” Cell 184, no. 25 (2021): 6101–6118, 10.1016/j.cell.2021.11.007. 34852236 PMC8671355

[70] W. Liu , W. You , Z. Lan , et al., “An Immune Cell Map of human Lung Adenocarcinoma Development Reveals an Anti‐tumoral Role of the Tfh‐dependent Tertiary Lymphoid Structure,” Cell Reports Medicine 5, no. 3 (2024): 101448, 10.1016/j.xcrm.2024.101448. 38458196 PMC10983046

[71] Z. Ji , J. Gao , Y. Xin , et al., “Fragrant TRPV3 Agonists Act as Titratable Organic Adjuvants to Amplify Antigen‐specific IgG Response,” Immunity & Inflammation 2, no. 1 (2026): 15, 10.1007/s44466-026-00033-5. 41797879 PMC12960365

[72] M. A. Morrissey , N. Kern , and R. D. Vale , “CD47 Ligation Repositions the Inhibitory Receptor SIRPA to Suppress Integrin Activation and Phagocytosis,” Immunity 53, no. 2 (2020): 290–302, 10.1016/j.immuni.2020.07.008. 32768386 PMC7453839

[73] Z. Zhou , M.‐J. M. Chen , Y. Luo , et al., “Tumor‐intrinsic SIRPA Promotes Sensitivity to Checkpoint Inhibition Immunotherapy in Melanoma,” Cancer Cell 40, no. 11 (2022): 1324–1340, 10.1016/j.ccell.2022.10.012. 36332624 PMC9669221

[74] N. Beyersdorf , X. Ding , J. K. Tietze , and T. Hanke , “Characterization of Mouse CD4 T Cell Subsets Defined by Expression of KLRG1,” European Journal of Immunology 37, no. 12 (2007): 3445–3454, 10.1002/eji.200737126. 18034419

[75] M. Feuerer , J. A. Hill , K. Kretschmer , H. von Boehmer , D. Mathis , and C. Benoist , “Genomic Definition of Multiple Ex Vivo Regulatory T Cell Subphenotypes,” Proceedings of the National Academy of Sciences 107, no. 13 (2010): 5919–5924, 10.1073/pnas.1002006107. PMC2851866 20231436

[76] L. M. Francisco , P. T. Sage , and A. H. Sharpe , “The PD‐1 pathway in tolerance and autoimmunity,” Immunological Reviews 236, no. 1 (2010): 219–242, 10.1111/j.1600-065X.2010.00923.x. 20636820 PMC2919275

[77] X. Wang , Y. Chai , Y. Quan , et al., “NPM1 inhibits Tumoral Antigen Presentation to Promote Immune Evasion and Tumor Progression,” Journal of Hematology & Oncology 17, no. 1 (2024): 97, 10.1186/s13045-024-01618-6. 39402629 PMC11479574

