# Supplementary material for: Tumor‐Specific Delivery of CD28 siRNA via Lyso‐PC C‐16 Modified Lipid Nanoparticles Overcomes Anti‐PD‐1 Resistance by Remodeling Tumor Microenvironment
Source: Adv Sci (Weinh). 2026 Jun 9;13(49):e76003. doi: 10.1002/advs.76003 (PMC13337025; doi:10.1002/advs.76003)
Supplement: Supplementary file 1 [file ADVS-13-e76003-s001.docx]

Supporting Information

Tumor-Specific Delivery of CD28 siRNA via Lyso-PC C-16 Modified Lipid Nanoparticles Overcomes Anti-PD-1 Resistance by Remodeling Tumor Microenvironment

*Yangyang Chai, Keyu Wang, Jiali Fang, Shaorui Jia, Yansong Shi, Wanfeng Gao, Xinpeng Liu, Jiaqiang Li, Zenghui Cui, Yazhi Qian, Xiaosu Chen, Dan Ding*, Xuetao Cao**

**Figure S1 Lands’ cycle activity in cancer cells**

(A) Dotplot showing related gene expression and Lands’ cycle activity in human breast cancers. (B) Dotplot showing related gene expression and Lands’ cycle activity in human TNBC. (C) Dotplot showing related gene expression and Lands’ cycle activity in human lung cancers. (D) Q-PCR analysis of *Lpcat1*, *Lpcat2*, *Lpcat3* and *Lpcat4* mRNA expression level in major cell subsets sorted from 4T1 tumor mass (n = 3).

**Figure S2 Silencing effiency of LPCAT enzymes in 4T1 cells**

(A-D) Western blot analysis of *Lpcat1* (A), *Lpcat2* (B), *Lpcat3* (C) and *Lpcat4* (D) silence efficacy in 4T1 cells.

**Figure S3 Gating strategy for LNP delivery efficiency detecting in splenocytes**

(A) Flow cytometry gating strategy for lymphoid cells LNP delivery efficiency detecting in splenocytes transfected with GFP mRNA loaded Dil-LNP. (B) Gating strategy for myeloid cells LNP delivery efficiency detecting in splenocytes transfected with GFP mRNA loaded Dil-LNP.

**Figure S4 Gating strategy for LNP delivery efficiency detecting *in vivo***

(A) Flow cytometry gating strategy for lymphoid cells and non-immune cells LNP delivery efficiency detecting in 4T1 tumors intratumorally injected with GFP mRNA loaded LNP. (B) Gating strategy for myeloid cells LNP delivery efficiency detecting in 4T1 tumors intratumorally injected with GFP mRNA loaded LNP.

**Figure S5 Gating strategy for cancer cell sorting from established mouse 4T1 tumor mass**

Flow cytometry gating strategy for cancer cell sorting and T cell CD28 detecting in 4T1 tumors receiving 3 doses LPC-LNP-Cd28 or COM-LNP-Cd28 intratumoral injection 10 days after tumor inoculation.

**Figure S6 Gating strategy for tumor microenvironment profiling**

(A) Flow cytometry gating strategy for lymphoid cell in tumor microenvironment. (B) Flow cytometry gating strategy for myeloid cell in tumor microenvironment.

**Figure S7 Immune cell percentage in 4T1 tumors receiving intratumoral LPC-LNP-Cd28 treatment**

(A) CD8^+^ T, (B) IFNγ^+^TNFα^+^CD8^+^ T, (C) Perforin^+^Granzyme B^+^CD8^+^ T and (D) IL2^+^CD8^+^ T analyzed by flow cytometry in 4T1 tumors intratumorally injected with indicated LNP containing 20 μg siRNA for 3 times (n = 6). (E) CD4^+^ T, (F) IFNγ^+^TNFα^+^CD4^+^ T, (G) IL2^+^CD4^+^ T and (H) Treg analyzed by flow cytometry in 4T1 tumors (n = 6). (I) NK and (J) Perforin^+^Granzyme B^+^NK analyzed by flow cytometry in 4T1 tumors (n = 6). (K) B cell, (L) cDC1, (M) cDC2, (N) Monocyte, (O) TAM and (P) Neutrophil analyzed by flow cytometry in 4T1 tumors (n = 6). Data are presented as mean ± Sem. Statistical significance was analysed by one-way ANOVA analysis with Tukey’s multiple comparison, *p < 0.05, **p < 0.01.

**Figure S8 Intratumoral *Cd28* siRNA delivery by LPC-LNP activates CD8^+^ T cell antitumor immune response in LLC tumors**

(A) CD8^+^ T, (B) IFNγ^+^TNFα^+^CD8^+^ T, (C) Perforin^+^Granzyme B^+^CD8^+^ T and (D) IL2^+^CD8^+^ T analyzed by flow cytometry in LLC tumors intratumorally injected with indicated LNP containing 20 μg siRNA for 3 times (n = 3). (E) CD4^+^ T, (F) IFNγ^+^TNFα^+^CD4^+^ T, (G) IL2^+^CD4^+^ T and (H) Treg analyzed by flow cytometry in LLC tumors (n = 3). (I) NK and (J) Perforin^+^Granzyme B^+^NK analyzed by flow cytometry in LLC tumors (n = 3). (K) B cell, (L) cDC1, (M) cDC2, (N) Monocyte, (O) TAM and (P) Neutrophil analyzed by flow cytometry in LLC tumors (n = 3). Data are presented as mean ± Sem. Statistical significance was analysed by one-way ANOVA analysis with Tukey’s multiple comparison, *p < 0.05, **p < 0.01.

**Figure S9 CyTOF analysis of tumor-infiltrating immune cells using lymphoid-centric panel for mouse 4T1 tumors receiving LPC-LNP-Cd28 and anti-PD-1 treatment**

(A) UMAP projection of CD45^+^ cells colored by PhenoGraph clusters from 4T1 tumors receiving LPC-LNP-Cd28 and αPD-1 combined treatment highlighting lymphoid cells by CyTOF from lymphoid centric panel. (B) Selected marker expression of CD45^+^ cells embedded in UMAP projection from lymphoid centric panel.

**Figure S10.** **Selected marker expression of lymphoid cells embedded in UMAP projection from lymphoid-centric panel.**

**Figure S11.** **Selected marker expression of CD45^+^ cells embedded in UMAP projection from myeloid-centric panel.**

**Table S1.** **Antibodies used for CyTOF lymphoid-centric panel**

| antibody | clone | label |
| --- | --- | --- |
| CD45 | 30-F11 | 89Y |
| CD44 | IM7 | 115In |
| CD11b | M1/70 | 139La |
| CD39 | 5F2 | 141Pr |
| CD11c | N418 | 142Nd |
| CD69 | H1.2F3 | 143Nd |
| CD4 | RM4-5 | 145Nd |
| PD-1 | RMP1-30 | 146Nd |
| TIM-3 | RMT3-23 | 147Sm |
| KLRG1 | 2F1 | 148Sm |
| CD19 | 6D5 | 149Sm |
| CD27 | LG.3A10 | 150Nd |
| CD25 | 3C7 | 151Eu |
| CD3e | 145-2C11 | 152Sm |
| CD8a | 53-6.7 | 153Eu |
| CTLA-4 | UC10-4B9 | 154Sm |
| CD86 | GL1 | 155Gd |
| CXCR3 | CXCR3-173 | 156Gd |
| TBET | 4B10 | 158Gd |
| RORγ | B2D | 159Tb |
| IFNγ | XMG1.2 | 160Gd |
| Perforin | S16009B | 161Dy |
| TNFα | MP6-XT22 | 162Dy |
| TIGIT | 1G9 | 163Dy |
| CD62L | MEL-14 | 164Dy |
| FoxP3 | FJK-16s | 165Ho |
| GATA-3 | L50-823 | 166Er |
| NKp46 | 29A1.4 | 167Er |
| ICOS | C398.4A | 168Er |
| EOMES | Dan11mag | 169Tm |
| TCF-1 | S33-966 | 170Er |
| Granzyme B | GB11 | 171Yb |
| Ki-67 | SolA15 | 172Yb |
| CD80 | 16-10A1 | 173Yb |
| LAG3 | C9B7W | 174Yb |
| CD127 | A7R34 | 175Lu |
| B220 | RA3-6B2 | 176Yb |
| MHC Ⅱ | M5/114.15.2 | 209Bi |

**Table S2.** **Antibodies used for CyTOF myeloid-centric panel**

| antibody | clone | label |
| --- | --- | --- |
| CD45 | 30-F11 | 89Y |
| CD11b | M1/70 | 139La |
| CD44 | IM7 | 115In |
| Ly6G | 1A8 | 141Pr |
| CD11c | N418 | 142Nd |
| CD24 | M1/69 | 143Nd |
| CD4 | RM4-5 | 145Nd |
| F4/80 | BM8 | 146Nd |
| XCR1 | ZET | 147Sm |
| CD103 | Polyclonal | 148Sm |
| CD19 | 6D5 | 149Sm |
| Ly6C | HK1.4 | 150Nd |
| CX3CR1 | SA011F11 | 151Eu |
| CD3e | 145-2C11 | 152Sm |
| PD-L1 | 10F.9G2 | 153Eu |
| CD38 | 90 | 154Sm |
| CD86 | GL1 | 155Gd |
| CCR7 | 4B12 | 156Gd |
| IL-10 | JES5-16E3 | 158Gd |
| CCR2 | 475301R | 159Tb |
| CD40 | 1C10 | 160Gd |
| CD163 | S15049I | 161Dy |
| SIRPα | P84 | 162Dy |
| CD206 | C068C2 | 163Dy |
| CD83 | Michel-19 | 164Dy |
| CD204 | 1F8C33 | 165Ho |
| Arginase 1 | Polyclonal | 166Er |
| NKp46 | 29A1.4 | 167Er |
| PD-1 | RMP1-30 | 168Er |
| SiglecH | 551 | 169Tm |
| SiglecF | S17007L | 171Yb |
| Ki-67 | SolA15 | 172Yb |
| CD80 | 16-10A1 | 173Yb |
| CD169 | 3D6.112 | 174Yb |
| CD8a | 53-6.7 | 175Lu |
| B220 | RA3-6B2 | 176Yb |
| MHC Ⅱ | M5/114.15.2 | 209Bi |
